# Invasive earthworms can change understory plant community traits and reduce plant functional diversity

**DOI:** 10.1016/j.isci.2024.109036

**Published:** 2024-01-29

**Authors:** Lise Thouvenot, Olga Ferlian, Dylan Craven, Edward A. Johnson, Johannes Köhler, Alfred Lochner, Julius Quosh, Anja Zeuner, Nico Eisenhauer

**Affiliations:** 1 German Centre for Integrative Biodiversity Research (iDiv) Halle-Jena-Leipzig, Puschstraße 4, 04103 Leipzig, Germany; 2 Leipzig University, Institute of Biology, Puschstraße 4, 04103 Leipzig, Germany; 3 GEMA Center for Genomics, Ecology & Environment, Universidad Mayor, Camino La Pirámide Huechuraba 5750, Santiago, Chile; 4 Data Observatory Foundation, Santiago, Chile; 5 Department Biological Sciences, University of Calgary, Calgary, AB T2N 1N4, Canada

**Keywords:** Ecology, Zoology, Plant ecology, Soil ecology

## Abstract

Among the most important impacts of biological invasions on biodiversity is biotic homogenization, which may further compromise key ecosystem processes. However, the extent to which they homogenize functional diversity and shift dominant ecological strategies of invaded communities remains uncertain. Here, we investigated changes in plant communities in a northern North American forest in response to invasive earthworms, by examining the taxonomic and functional diversity of the plant community and soil ecosystem functions. We found that although plant taxonomic diversity did not change in response to invasive earthworms, they modified the dominance structure of plant functional groups. Invasive earthworms promoted the dominance of fast-growing plants at the expense of slow-growing ones. Moreover, earthworms decreased plant functional diversity, which coincided with changes in abiotic and biotic soil properties. Our study reveals that invasive earthworms erode multiple biodiversity facets of invaded forests, with potential cascading effects on ecosystem functioning.

## Highlights

• Invasive earthworms reduced the diversity of woody plant species
• Plant functional diversity and ecological strategies shifted in invaded area
• Invasive earthworms functionally homogenized plant community
• Earthworms reduced litter thickness, soil microbial activity, and nutrients

## Graphical abstract

**Figure:**
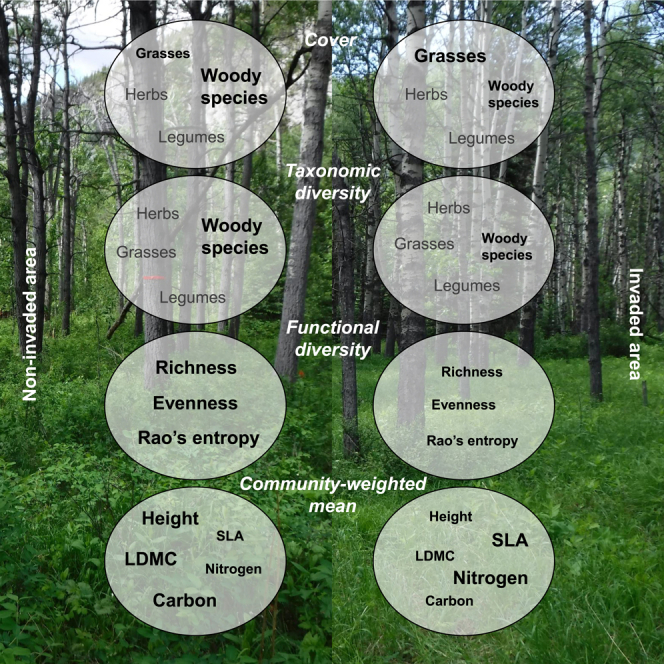

## Introduction

There is growing evidence that ecosystems and biodiversity face multiple threats.^1^^,^^2^ This body of research has highlighted the major role of direct exploitation, climate change, land use change and degradation, pollution, and biological invasions in driving the loss of biodiversity and the disruption of associated ecosystem functions.^1^^,^^3^^,^^4^^,^^5^^,^^6^^,^^7^ Biotic homogenization is one of the most prominent impacts of biological invasions on biodiversity, which may further compromise key ecosystem processes.^8^^,^^9^ It consists of the increase of the genetic, taxonomic, and/or functional similarity between communities.^8^^,^^10^ While there is growing evidence that biological invasions homogenize native communities taxonomically, whether invaded communities become more functionally similar is uncertain,^8^^,^^11^ which is in line with the poor use of the functional diversity indices in the context of biological invasions.^12^

Biotic homogenization can result from the increase in the number of species shared between communities: when the invader of the same trophic-level as the community establishes at several locations, it increases community similarity.^8^^,^^13^ The homogenization process can also happen when the establishment of the invasive species (from the same trophic-level or not as the one of the communities considered) induces a similar species loss across the communities, resulting in greater compositional similarity.^8^^,^^13^ In this case, we could also expect biotic differentiation with an increase in dissimilarity, if different species are lost across communities for example.^14^ The ecological mechanisms that underlie biotic homogenization due to invasive species establishment and spread in native ecosystems can be diverse. Invasive species can, for example, directly affect native species via competitive^15^^,^^16^ or feeding interactions.^17^^,^^18^ They also indirectly affect native species via other organisms^19^^,^^20^^,^^21^ or by altering environmental conditions.^22^ Consequently, invasive species can shape the future of the invaded native communities by selecting for particular species of the native community thus giving to the community a novel combination of functional trait values,^12^ and probably facilitating the invasion of other species.^23^ Shifts in species composition, dominance structure, and trait diversity and composition due to biological invasions are likely to impact ecosystem functioning,^24^ following the mass ratio hypothesis.^25^

While the impacts of aboveground invasive species are well known, those of belowground invasive invertebrates, such as earthworms, are underappreciated.^13^ However, they could be more significant when invasive species are ecosystem engineers.^26^^,^^27^ Invasive earthworms, for example, can be particularly impactful to native biodiversity and ecosystems that previously lacked native earthworms, such as in most northern North American forests^28^^,^^29^^,^^30^^,^^31^ and Arctic regions.^32^ Recent studies showed that invasive earthworms affect soil microorganisms, soil invertebrates,^33^^,^^34^^,^^35^^,^^36^ as well as native plant communities,^37^^,^^38^ and soil surface/vegetation-dwelling invertebrates.^31^^,^^36^^,^^39^ The ecological mechanisms behind their impacts are numerous, and the direction and magnitude of the effects vary with earthworm diversity and community composition.^35^^,^^38^^,^^40^

First, because of their castings, litter feeding, soil burrowing and mixing activities, invasive earthworms can alter the soil structure, its stability, and the distribution of soil water and nutrients.^40^^,^^41^^,^^42^^,^^43^^,^^44^^,^^45^^,^^46^^,^^47^^,^^48^ Second, invasive earthworms can directly affect plant communities via multiple paths. For example, shifts in plant community composition and taxonomic diversity associated with the presence of invasive earthworms^49^^,^^50^^,^^51^^,^^52^ can be explained by their effects on seed bank composition, as well as seed germination, survival, and development.^37^^,^^49^^,^^53^^,^^54^^,^^55^^,^^56^^,^^57^^,^^58^ Moreover, invasive earthworms do not only affect the initial stages of plant community assembly (or re-assembly). Their effects on plant communities could also be explained by their impacts on plant traits and biomass.^59^^,^^60^ However, their impacts on trait diversity and composition, while hypothesized, have yet to be examined. Earthworms have been shown to impact traits linked to plant development and resource uptake, such as plant height,^61^ nutrient content,^48^ root growth,^32^^,^^62^ and mycorrhizal association,^63^ but also traits linked to plant vegetative reproduction, such as the number of culms.^64^ Plant trait responses to earthworm presence are context dependent, depending on plant species and functional group identity.^60^^,^^65^^,^^66^ Indeed, although the underlying ecological mechanisms are understudied, grasses seem to benefit more from earthworm activity than herbs,^60^ and dominance of grass species is commonly observed in the presence of invasive earthworms.^38^^,^^67^

We, therefore, hypothesize that plant species that are more efficient in taking up resources under conditions of higher resource availability will dominate areas with invasive earthworms which, in turn, will alter habitat conditions. The abundance of fast, or ‘acquisitive’ plant species,^68^^,^^69^ which typically have high specific leaf area, nitrogen content, and specific root length, as well as low leaf dry matter and carbon content (like many grass species), would be higher in invaded areas. Indeed, most grass species possess traits associated with fast growth and resource uptake.^70^^,^^71^ This selection process linked to the activity of invasive earthworms is expected to lead to a decrease in taxonomic diversity and an increase in plant species functional similarity in invaded areas. As the dominance of fast-growing plant species would increase with the presence of invasive earthworms, we expect to observe a shift in trait composition. Overall, the community-weighted mean of leaf nitrogen content and specific leaf area would be higher, while the community-weighted mean leaf dry matter content would be lower in plant communities in invaded areas than in non-invaded areas. This shift in trait composition would coincide with a decrease in the functional richness, dispersion, and evenness of the plant community.

Our study aims to give new insights into plant community responses to earthworm invasion by evaluating the effect of invasive earthworms on plant functional diversity, and multiple facets of biotic homogenization.

By studying the taxonomic and functional diversity responses of plant communities in a northern North American aspen forest, we disentangle the ecological mechanisms behind changes in understory plant communities and ecosystem functions linked to invasive earthworm presence. We expected invasive earthworms to lead to (1) a biotic homogenization of the plant community, with lower taxonomic and functional diversity in invaded area than in uninvaded area, and to (2) a shift in trait composition, with invaded area dominated by plant species with acquisitive strategies compared to uninvaded area. We also expect to observe (3) a change in ecosystem functions, such as litter decomposition, soil nutrient content, and soil microbial biomass and activity in the invaded area.

## Results

### Effects of invasive earthworms on plant taxonomic diversity at the community and plant functional group levels

**Figure 1:**
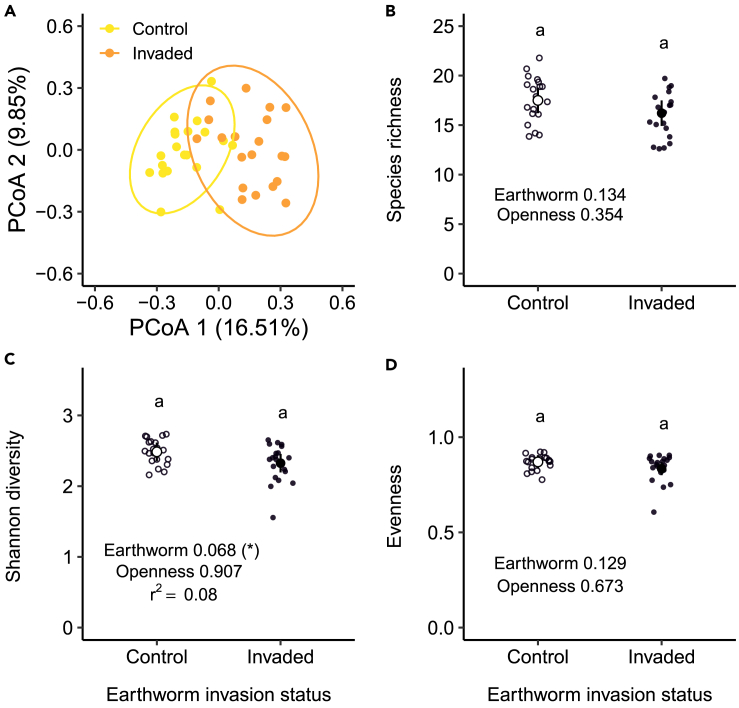
Effects of invasive earthworms on plant taxonomic diversity Principal coordinate analysis showing differences in plant community composition (A) (control (yellow) vs. invaded (orange) area), with ellipses showing the 95% confidence intervals), and the effects of invasive earthworms on plant species richness (B) Shannon diversity (C), and plant evenness (D) measured in the Barrier Lake North forest (control (○) vs. invaded (•) area). Estimated marginal means and 95% confidence intervals are shown (after being back-transformed when necessary), while data points are included in the background. The p values are based on linear models, and the adjusted r^2^ of the models are shown when at least one factor was (even marginally) significant. Significance codes: (∗) <0.10. See also Table S2.

While we observed a homogeneous dispersion among areas, i.e., the average Bray-Curtis distance to the center of the invaded and control areas were similar (F = 0.19; p = 0.66), the PERMANOVA showed that plant community composition differed between the non-invaded and invaded earthworm areas (F = 4.87; p < 0.001, Figure 1A), with the earthworm invasion status explaining 11% of the variation in plant community composition (r^2^ = 0.11).

However, we did not observe any significant changes in plant species richness (F = 2.35, p = 0.13, Figure 1B), or plant evenness (F = 2.41 p = 0.13, Figure 1D) in response to earthworm invasion or canopy openness (F = 0.88, p = 0.35 and F = 0.18, p = 0.67, respectively) at the community level. Only Shannon diversity slightly decreased by 6.4% (F = 3.54, p = 0.07) in the presence of invasive earthworms (Figure 1C; Tables S2 and S8), but was not affected by canopy openness (F = 0.014, p = 0.91).

**Figure 2:**
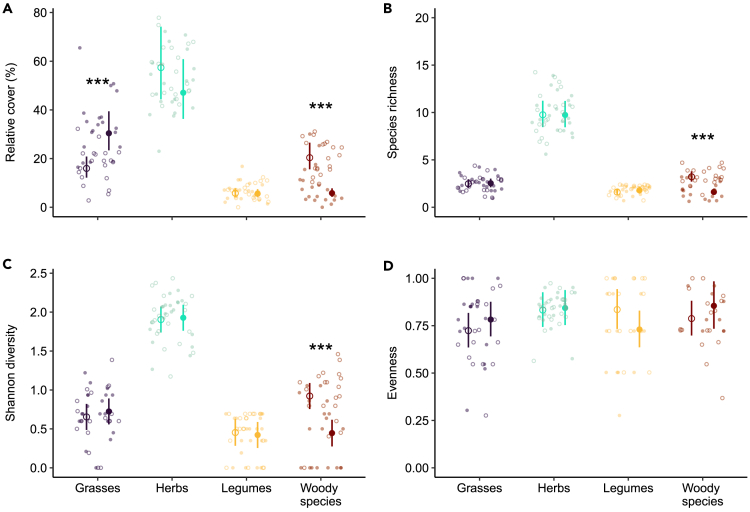
Effects of invasive earthworms on plant functional group diversity and cover Relative cover (A), richness (B) Shannon diversity (C) and evenness (D) of the different plant functional groups measured in the Barrier Lake North forest according to the earthworm invasion status (control (○) vs. invaded (•) area). Estimated marginal means and 95% confidence intervals are shown (after being back-transformed, when necessary), while data points are included in the background. The results of the post hoc tests performed when the interactions between earthworm invasion status and plant functional groups were significant in the linear models are displayed with stars that show significant differences between earthworm invasion status for the specific functional group. Significance codes: ∗∗∗ <0.001. See also Table S3.

In contrast, we observed changes in diversity and plant cover at the plant functional group level, with earthworms affecting mainly grasses positively and woody plants negatively (Figure 2; Tables S3 and S8). Indeed, relative cover varied across plant functional groups (F = 132.84, p < 0.001), earthworm invasion status (F = 4.34, p = 0.04), and their interaction (F = 22.20, p < 0.001), and marginally in response to canopy openness (F = 3.41, p = 0.07). We found that in the presence of earthworms the cover of woody plants was reduced by 71.5%, while that of grasses increased by 90% (Figure 2A). Thus, the relative cover of grasses and woody species, which were on average ∼16% and ∼20% cover (back-transformed estimated marginal means) in plots of the control area, respectively, shifted to ∼30% and ∼6% of cover on average per plots (back-transformed estimated marginal means), respectively, in invaded area.

Similarly, our study shows that the species richness (Figure 2B) and Shannon diversity (Figure 2C) differed between plant functional groups (F = 251.56, p < 0.001, and F = 170.15, p < 0.001 respectively), and varied across earthworm invasion status (F = 4.54, p = 0.03 and F = 2.84, p = 0.09, respectively), and their interaction (F = 10.45, p < 0.001 and F = 5.99, p < 0.001, respectively), but not in response to canopy openness (F = 0.33, p = 0.56 and F = 0.28, p = 0.60 respectively). In invaded area, species richness and Shannon diversity of woody plants declined by 49.8% and by 51.6%, respectively. While we observed an average woody plant richness of ∼3.2 species and a Shannon diversity of 0.92 in the control area, species richness was about ∼1.6 and the Shannon diversity of 0.44 in the invaded area. However, we did not observe any significant differences across plant functional groups (F = 1.71, p = 0.17), earthworm invasion status (F = 0.05, p = 0.81), their interaction (F = 1.67, p = 0.18), or canopy openness (F = 0.002, p = 0.96) for plant functional group evenness (Figure 2D).

### Effects of invasive earthworms on plant functional diversity and plant functional trait composition

**Figure 3:**
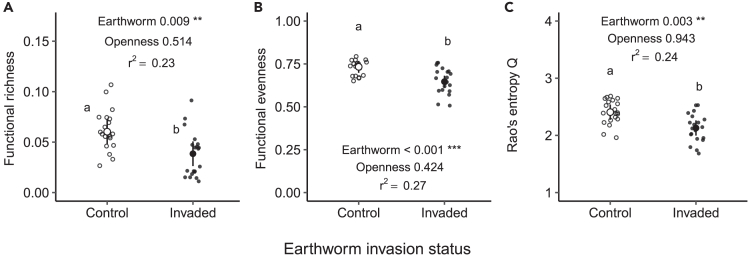
Effects of invasive earthworms on plant functional diversity Effects of invasive earthworms on the standardized functional richness (A), functional evenness (B), and Rao’s entropy index (C) of the plant community of the Barrier Lake North forest according to the earthworm invasion status (control (○) vs. invaded (•) area). Estimated marginal means and 95% confidence intervals are shown, while data points are included in the background. The p values are based on linear models, and the adjusted r^2^ of the models are shown when at least one factor was significant. The different letters show significant differences between earthworm invasion status. Significance codes: ∗∗∗ <0.001; ∗∗ <0.01; ∗<0.05. See also Tables S4, S9, and S12.

**Figure 4:**
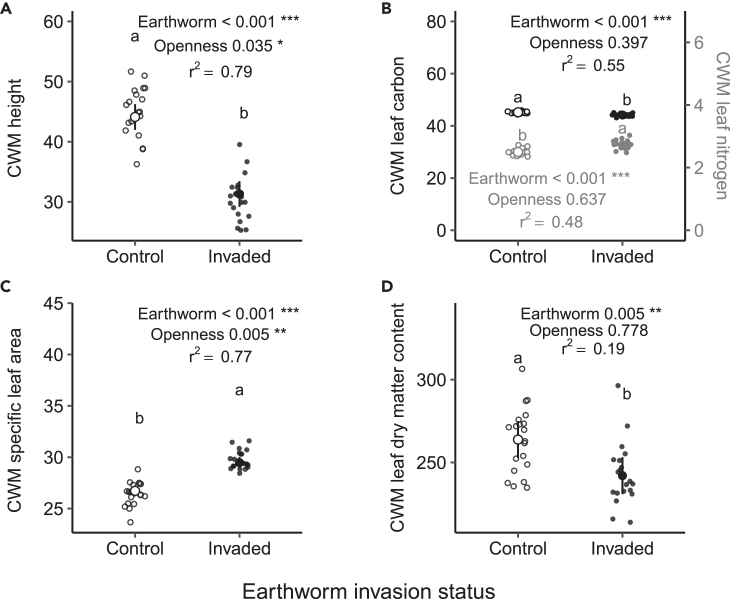
Effects of invasive earthworms on plant community traits Community weighted mean (CWM) of the height (A), the leaf carbon and nitrogen content (B), the specific leaf area (C) and the leaf dry matter content (D) of the plant community of the Barrier Lake North forest according to the earthworm invasion status (control (○) vs. invaded (•) area). Estimated marginal means and 95% confidence intervals are shown, while data points are included in the background. The p values are based on linear models, and the adjusted r^2^ of the models are shown when at least one factor was significant. The different letters show significant differences between earthworm invasion status. Significance codes: ∗∗∗ <0.001; ∗∗ <0.01; ∗<0.05. See also Tables S5, S9, and S12–S15.

Invasive earthworms significantly altered plant functional diversity (Figure 3; Tables S4 and S9) and plant trait composition (Figure 4; Tables S5 and S9). Functional richness was reduced by 36.2% in the invaded area (F = 7.54, p = 0.009, Figure 3A). Moreover, a ∼12% reduction of the functional evenness (−11.8%) and Rao’s entropy (−11.5%) was observed in the earthworm-invaded area (F = 14.72, p < 0.001, Figure 3B and F = 10.27, p = 0.003; Figure 3C respectively). Moreover, none of these indices were significantly affected by canopy openness (F = 0.43, p = 0.51; F = 0.65, p = 0.42 and F = 0.005, p = 0.94, respectively).

We also found that plant community trait composition changed in the earthworm-invaded area (Figure 4). The CWM of height was ∼13 cm lower on average in the invaded area than in the control area (percentage of change: −29%, F = 84.6, p < 0.001, Figure 4A). Leaf trait values also shifted in response to earthworm invasion: the CWM of SLA increased by 10.3% (F = 62.69, p < 0.001, Figure 4C), and the CWM of SLA was 2.7 mm^2^ mg^−1^ higher in the invaded area than in the control area. By contrast, the CWM of LDMC decreased by 8.2% (F = 8.96, p = 0.005, Figure 4D) in the invaded area: the CWM of LDMC was 21.7 mg g^−1^ lower in the invaded area than in the control area. Moreover, the CWM of leaf carbon was reduced by 2.3% (F = 40.42, p < 0.001, Figure 4B) and reached an averaged content of 45.2% of carbon in the leaves of the communities of the invaded area, while the CWM of leaf nitrogen increased by 8.9% (F = 24.94, p < 0.001, Figure 4B) to reach an average amount of 2.72% of nitrogen in leaves in the invaded area. Canopy openness increased the CWM of height (estimate _=_ 0.23, CI_95% =_[0.02, 0.44], F = 4.79, p = 0.035), and decreased the CWM of SLA (estimate _=_ −0.8, CI_95% =_[-0.13, −0.03], F = 8.76, p = 0.005), but did not significantly affect the CWM of LDMC (F = 0.08, p = 0.78), leaf carbon (F = 0.73, p = 0.40), and nitrogen content (F = 0.23, p = 0.64).

**Figure 5:**
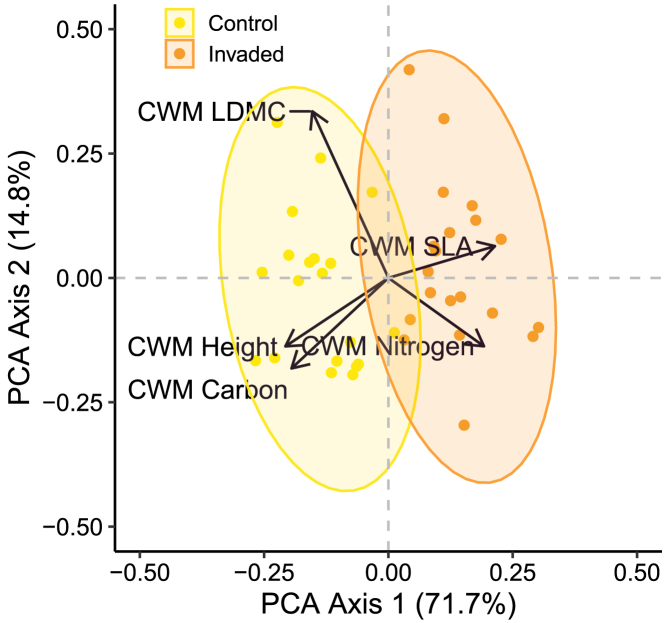
Community weighted mean traits distribution characterizing the earthworm invasion areas Community weighted mean (CWM) of the height, the leaf carbon and nitrogen content, the specific leaf area (SLA) and the leaf dry matter content (LDMC) of the plant community of the Barrier Lake North forest, displayed using a Principal Component Analysis (PCA) to characterize the earthworm invasion areas. CWMs were centered and scaled, and the percentage of variance explained for each axis is in brackets. Contributions of CWMs to each axis can be found in Table S10, and the fit of ecosystem functions to axes in Table S11 and Figure S2.

Moreover, the PCA reveals a shift in dominant plant strategies in response to earthworm invasion (Figure 5), from communities dominated by slow plant species with low nitrogen content, and SLA to those dominated by fast plant species with high nitrogen content and SLA, but low LDMC.

### Effects of invasive earthworms on soil abiotic properties and ecosystem functions

**Figure 6:**
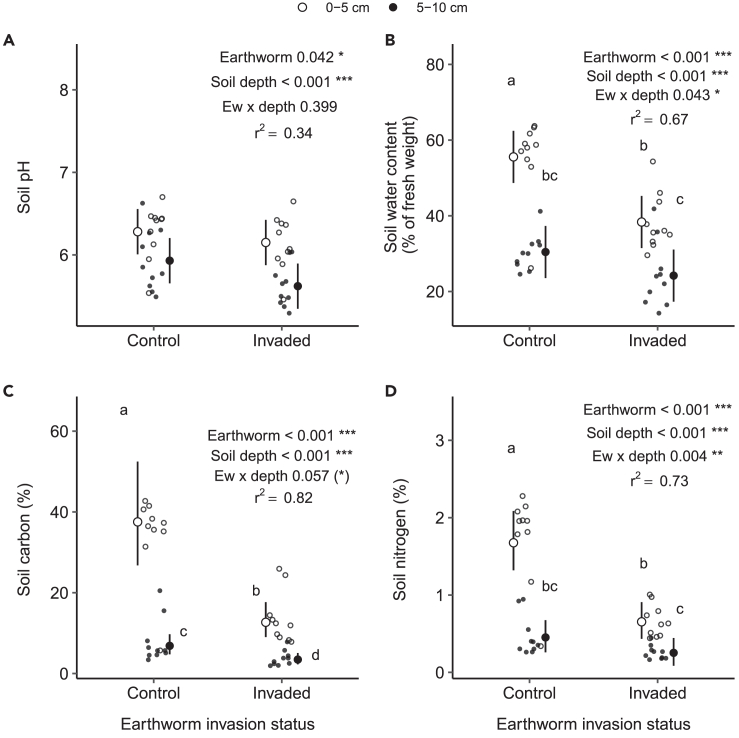
Effects of invasive earthworms on soil abiotic parameters Soil pH (A), water content (B), carbon (C) and nitrogen (D) measured in the Barrier Lake North forest according to the earthworm invasion status and soil depth (0–5 cm (○) vs. 5–10 cm (•)). Estimated marginal means and 95% confidence intervals are shown (after being back-transformed when necessary), while data points are included in the background. The p values and r^2^ are based on linear models. Letters correspond to the results of post hoc tests performed when the interaction between earthworm and soil depth were significant: different letters show significant differences between soil depth and earthworm invasion areas. Significance codes: ∗∗∗ <0.001; ∗∗ <0.01; ∗<0.05; (∗) <0.10. See also Tables S6, S8, S14, and S15.

Some soil properties and ecosystems functions like the soil water, nitrogen and carbon content, as well as microbial biomass, respiration, and activity shifted in the earthworm-invaded area, especially in the upper (0–5cm) soil layer (Figure S2; Table S11). Indeed, the pH of the soil was more acidic in the invaded than in the control area (pH of 5.9 in the invaded area vs. 6.1 in the control area; percentage of change: −3.6%, F = 4.45, p = 0.04, Figure 6A), and in the deeper soil layer than in the upper one (pH of 5.8 vs. 6.2, respectively; percentage of change: −7.1%, F = 17.81, p < 0.001). Moreover, soil contained 54.4% less carbon in the invaded area than in the control area (earthworm effect: F = 24.03, p < 0.001) and 75.4% less in the deeper soil layer than in the upper soil layer (soil depth effect: F = 75.98, p < 0.001). However, the interaction between soil depth and invasive earthworm status was not significant for these two soil parameters (pH: F = 0.73, p = 0.40, and soil carbon: F = 1.03, p = 0.32). Removing one outlier in the soil carbon data lead to a marginally significant effect of the interaction of earthworm invasion status and soil depth (F = 3.87, p = 0.06, Figure 6C; Tables S6 and S8), with a negative effect of invasive earthworms being more pronounced on soil carbon in the upper soil layer as compared to the deeper soil layer. Moreover, the soil water content and nitrogen content also decreased in invaded area (−27.3%, F = 20.08, p < 0.001, Figure 6B and −54.7%, F = 33.55, p < 0.001, Figure 6D, respectively), by soil depth (−41.8%, F = 56.42, p < 0.001, and −68.4% F = 67.11, p < 0.001, respectively), and were significantly affected by the interaction between the two factors (F = 4.39, p = 0.04 and F = 9.43, p = 0.004, respectively). More specifically, while the presence of invasive earthworms did not affect the nitrogen content in the deeper soil layer significantly, we observed a decrease by ∼61% in the upper soil layer in the earthworm-invaded area: the nitrogen content reached only 0.6% in the invaded area, while having an average value of 1.7% in the control area in this layer. In a similar way, the soil water content did not change in the deeper soil layer due to earthworms, but while the percentage of water was ∼55.6% in the control area, it decreased to 38.3% in the invaded area in the upper soil layer, which represents an approximate decrease of ∼31% of water content.

**Figure 7:**
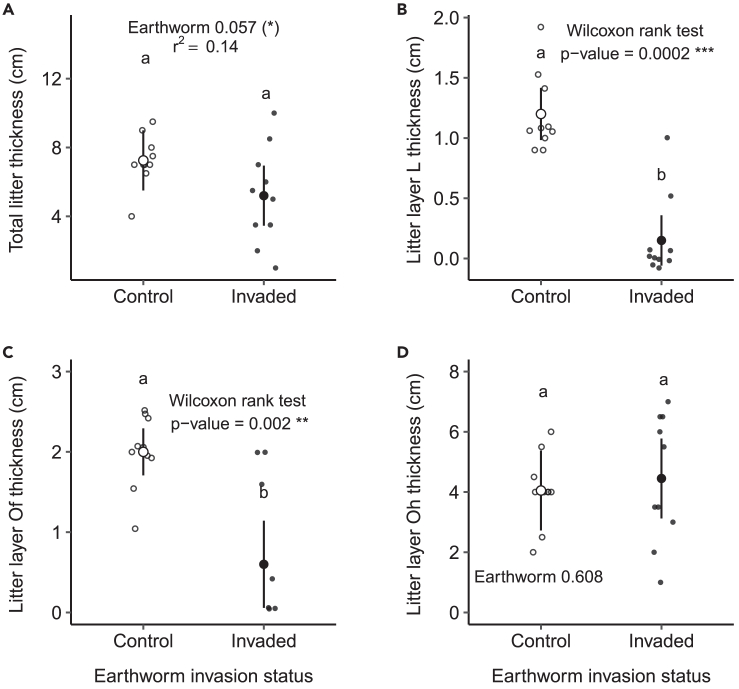
Effects of invasive earthworms on litter Total litter thickness (A), L litter thickness (B), Of litter thickness (C) and Oh litter thickness (D) measured in the Barrier Lake North forest according to the earthworm invasion status (control (○) vs. invaded (•) area). Estimated marginal means or means (for the L and Of litter thickness) and 95% confidence intervals are shown (after being back-transformed when necessary), while data points are included in the background. The p values and r^2^ are based on linear models, or on non-parametric Wilcoxon tests when necessary. The different letters show significant differences between earthworm invasion status. Significance codes: ∗∗∗ <0.001; ∗∗ <0.01; (∗) <0.10. See also Tables S7 and S13.

In addition, invasive earthworms marginally reduced total litter layer thickness by 28.3% (F = 4.12, p = 0.06, Figure 7A; Table S7): the thickness was on average ∼2 cm thinner in the invaded area than in the control area. This was probably largely driven by the negative effects of invasive earthworm presence on the thickness of the L litter (−87.5%, Wilcoxon test; W = 96.5, p < 0.001, Figure 7B) and Of litter layers (−70%, Wilcoxon test; W = 89.5, p = 0.002, Figure 7C). Litter thickness in the control area was on average ∼1.2 cm (L layer) and ∼2 cm (Of layer), respectively, and were reduced to a thickness of ∼0.15 cm and ∼0.6 cm, respectively, in the invaded area. The thickness of the Oh layer did not change significantly between control and invaded areas (F = 0.27, p = 0.61, Figure 7D).

**Figure 8:**
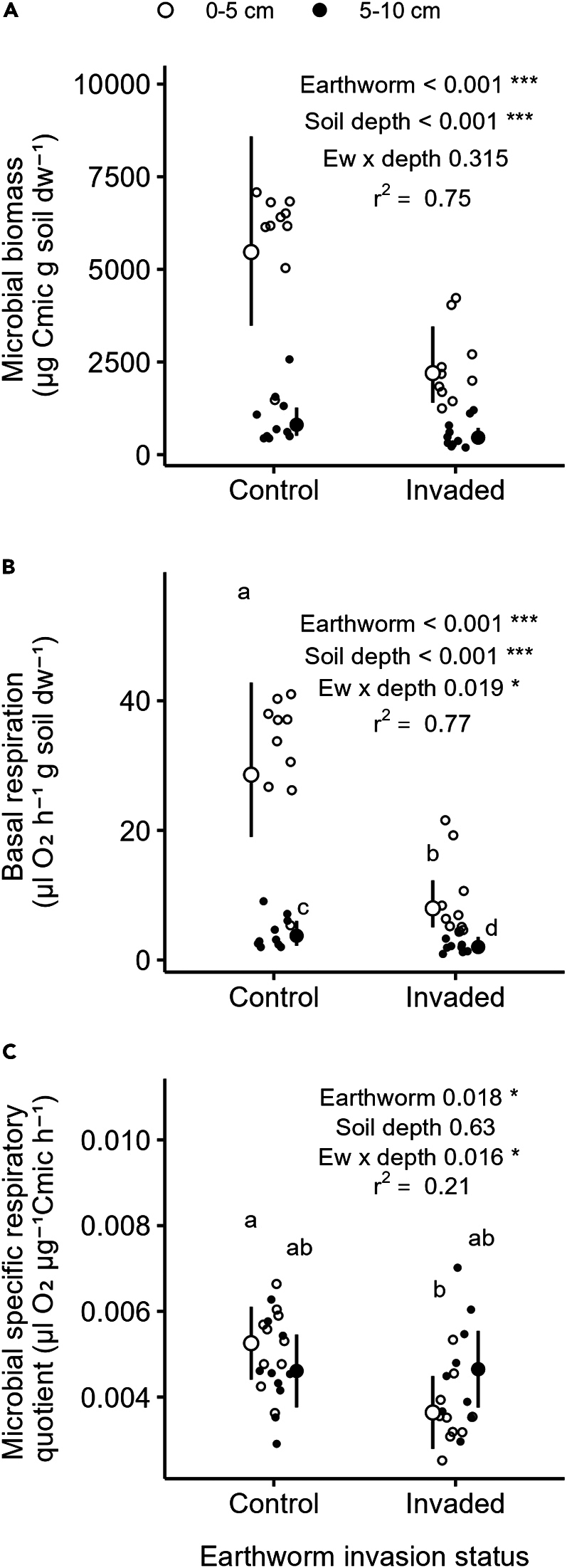
Effects of invasive earthworms on soil microbial activity Soil microbial biomass (A), basal respiration (B) and microbial specific respiratory quotient (C) measured in the Barrier Lake North according to the earthworm invasion status and soil depth (0–5 cm (○) vs. 5–10 cm (•)). Estimated marginal means and 95% confidence intervals are shown (after being back-transformed when necessary), while data points are included in the background. The p values and r^2^ are based on linear models. Letters correspond to the results of post hoc tests performed when the interaction between earthworm invasion status and soil depth were significant: different letters show significant differences between soil depth and earthworm invasion status. Significance codes: ∗∗∗ <0.001; ∗<0.05. See also Tables S6, S14, and S15.

Moreover, soil microbial biomass decreased significantly (−52%) in the earthworm-invaded area (F = 18.34, p < 0.001, Figure 8A; Table S6) and across soil depths (−82.4% from the top to the deeper soil layer, F = 102.51, p < 0.001), but did not change in response to the interaction between these factors (F = 1.04, p = 0.31). Soil basal respiration also decreased significantly by 33.3% in the presence of invasive earthworms (F = 30.47, p < 0.001, Figure 8B) and by 29.1% across soil depths (soil depth: F = 95.02, p < 0.001). The significant interaction effect between the presence of invasive earthworms and soil depth (F = 6.03, p = 0.02) was probably due to a stronger, negative effect of invasive earthworm presence on soil basal respiration in the upper soil layer than in the lower soil layer. Soil basal respiration was on average 28.6 μL O_2_ h^−1^ g^−1^ in the control plots and reached on average 8 μL O_2_ h^−1^ g^−1^ in the invaded area (−72.1%), while in the lower soil layer, it was on average 3.7 μL O_2_ h^−1^ g^−1^ in the control area and reached 2 μL O_2_ h^−1^ g^−1^ in the invaded area (−46.1%).The soil microbial specific respiratory quotient was significantly reduced (−16%) in the invaded area (F = 6.17, p = 0.018, Figure 8C), but this effect depended on soil depth, resulting in a significant interaction between invasive earthworms and soil depth (F = 6.45, p = 0.016). More specifically, the microbial specific respiratory quotient of the upper soil layer significantly decreased from 0.005 μL O_2_ μg^−1^ Cmic h^−1^ in the control area to 0.0036 μL O_2_ μg^−1^ Cmic h^−1^ in the invaded area, thus corresponding to a decrease of 30.7%, in the upper soil layer, but it did not change significantly in the lower soil layer. However, soil depth alone had no significant effect on the microbial specific respiratory quotient (F = 0.24, p = 0.63).

## Discussion

### Invasive earthworm presence is associated with functional homogenization of the plant community

We expected a negative effect of invasive earthworm presence on taxonomic plant diversity, with a taxonomic simplification of the community in the earthworm-invaded area. This decline in taxonomic diversity would be mainly driven by a decrease in the diversity of plant functional groups, particularly woody and herbaceous species. However, counter to our expectations, our results did not show any significant decline in the taxonomic diversity in the presence of invasive earthworms nor an increase in plant taxonomic similarity, despite a change in plant community composition. While some previous studies have shown positive or no relationships between taxonomic plant diversity and invasive earthworms,^49^^,^^55^ most studies showed a decrease in native plant species richness with an increasing biomass/abundance of invasive earthworms.^49^^,^^52^^,^^72^ Moreover, the meta-analysis by Craven et al.^38^ reported an overall decline in plant Shannon diversity but also showed that non-significant plant taxonomic diversity responses could mask contrasting changes in native and non-native plant species.^38^ Similarly, we found that the absence of changes in the taxonomic diversity at the plant community level in the invaded area, does not reflect the response observed at the plant functional group level, which implies that assessing community changes in response to earthworm invasion by measuring diversity indices at the community level may mask responses of particular species or plant functional groups to earthworms.

Indeed, when we zoomed in on the responses of plant functional group diversity, we found that in the earthworm-invaded area, the cover of grasses increased, while the cover, species richness, and Shannon diversity of woody plants decreased. Similar results with changes in plant community richness due to particular species or plant functional groups have been reported in the literature.^51^^,^^52^^,^^73^^,^^74^ For example, Drouin et al.^52^ and Alexander et al.^74^ showed that grass abundance/cover was higher in the presence of a high density of invasive earthworms, while the cover of herbs from the *Asteraceae* or *Violaceae* families^74^ or woody plants,^51^ decreased with earthworm invasion. However, the changes we observed were not related to an increase in the dominance of a particular plant species or functional group, as evenness was not significantly affected at the community or functional group levels. Thus, our results show that invasive earthworms can have significant effects on plant community structure and functional group composition without leading to an increase in the taxonomic similarity of the community.

Moreover, although we did not observe taxonomic homogenization of the community, we found a functional homogenization of the communities in the earthworm-invaded area, with lower functional diversity in invaded area than in the control area. More specifically, we observed a significant reduction in functional richness in the presence of invasive earthworms. This change could be related to the decrease in the richness of woody species, which could be due to multiple mechanisms. Invasive earthworms could either directly affect seed germination via their burrowing activities,^54^ or they could indirectly affect plant establishment and development by altering the soil nutrient content of the soil, thus promoting the development/establishment of more resource-acquisitive plant species.^38^^,^^65^ The decrease in the richness of woody species and/or the increase in the cover of the resource acquisitive species in invaded communities would be consistent with the decrease in the functional evenness and Rao’s entropy, which were also negatively affected and respectively slightly correlated to the CWM LDMC and CWM carbon in the invaded area (Table S12). The decrease in the functional evenness in the earthworm-invaded area suggests that plant species and their abundances were less regularly distributed in the functional space than in the non-invaded areas. This reduction in functional evenness indicates species packing, with more groups of functionally similar species present in the invaded area, possibly suggesting that resources are probably underused.^75^^,^^76^^,^^77^^,^^78^ This finding is further confirmed by the decrease in Rao’s entropy in the presence of invasive earthworms, indicating that plant species are more functionally similar in the earthworm-invaded area than in the control area. Our results thus show an increase in the functional similarity of the plant species and, thus a functional homogenization of the plant communities in the presence of invasive earthworms.

Invasive species effects on the functional diversity of native communities and functional homogenization are still understudied, and even more so when the invader does not belong to the same trophic level. It is thus difficult to compare our results to existing literature. To our knowledge, this study is the first to investigate changes in understory plant functional diversity in the context of earthworm invasion, and the few examples of biological invasion effects on functional diversity show mixed results. For example, Allen et al.^79^ found that the invasive plant species *Sonchus arvensis* and *Cirsium arvense* had a positive effect on the functional divergence and evenness of boreal wetland plant communities. In contrast, Hejda and de Bello^80^ found that invasive plants decreased the functional richness and evenness of plant communities, and Chabrerie et al.^81^ showed that *Prunus serotina* induced a decrease of Rao’s quadratic entropy in the invaded understory plant communities. Moreover, Wong et al.^82^ showed that the ant species (also considered as an ecosystem engineer^26^) *Solenopsis invicta,* acts as an environmental filter on native ant communities of tropical grasslands, with a decrease of the functional divergence and Rao’s entropy in invaded communities, while functional richness and evenness were not affected by the presence of the invasive ants. These mixed results can be due to diverse causes. For example, the functional diversity responses of the community to invasion appear to depend on the time since invasive species establishment, the intensity/degree of invasion, and/or the (functional) diversity of the invasive species community.^83^^,^^84^^,^^85^ It is thus necessary to further investigate the different responses of the native plant functional diversity when considering invasive earthworm abundance, community composition, or its functional diversity instead of the presence/absence of invasive earthworms only.

### Invasive earthworms change plant trait composition with potential implications for ecosystem functioning

Shifts in trait composition might have contributed to the observed changes in ecosystem functions, with non-invaded areas with plant communities dominated by slow plant species (i.e., high LDMC, carbon content, and low SLA) being associated with the highest soil water and nutrient content, microbial composition and activity (Table S11; Figure S2). In the present study, changes in plant functional group diversity and composition coincided with overall changes in trait composition, with invaded area dominated by plant species with resource acquisitive strategies, i.e., traits that lead to a more efficient nutrient uptake from the soil, compared to the non-invaded area. Indeed, the presence of invasive earthworms increased the CWM of specific leaf area and leaf nitrogen content and simultaneously decreased the CWM of height, leaf dry matter content, and leaf carbon content, which could potentially have contributed to the slight decrease in the total litter layer thickness. Indeed, low leaf dry matter content, high specific leaf area, and leaf nitrogen content are known to accelerate decomposition.^86^^,^^87^^,^^88^ Thus, litter decomposition might be faster following earthworm invasion, as understory plant litter will be more readily decomposed. However, the contribution of the understory litter to total litter thickness needs to be investigated, as we would expect litter thickness to be driven by tree leaves in forests, which would explain the general absence of correlations between trait CWMs and the litter thickness in control and earthworm-invaded areas (Table S13). For example, Holdsworth et al.,^89^ Hobbie et al.,^90^ as well as Côté and Fyles^91^ showed that the composition and/or the quality of the tree species litter affected the rate of litter decomposition, with a faster disappearance of litter with high calcium content,^90^ and of high litter quality in the presence of invasive earthworms.^89^

Moreover, in our study, trait CWMs were weakly correlated with soil properties and ecosystem functions (Tables S14 and S15), and, surprisingly, we observed more significant correlations between trait CWMs and ecosystem properties in the deeper soil layer (5–10 cm; Table S15) than in the upper (0–5 cm) soil layer. For example, the CWM of LDMC was negatively correlated with the pH of the upper soil layer and to the nutrient content (C and N) as well as the basal respiration of the deeper soil layer of the earthworm-invaded area, while it was positively correlated with soil nitrogen and water content, as well as basal respiration and microbial biomass of the deeper soil layer in the control area. The negative relationship between the CWM of LDMC and the nutrient and water content in soil in the earthworm-invaded area could be explained by an increase in their leaching, and/or by changes in the resource uptake strategies of the plant community due to the reduction in wood plant diversity or the increase in grass cover in the presence of earthworms. These relationships are speculative, and we acknowledge that a higher number of replicates would be required in future studies to better disentangle the links between plant community traits/diversity and the soil and ecosystem parameters via multivariate analyses, such as structural equation modeling. We thus stress the need of investigating these relationships in further studies, by taking into account, among others, tree litter quality and amount, the priming effect, as well as the plant cover and litter microclimate effects on soil microbes that could reveal the mechanisms behind shifts in ecosystem functions and properties due to invasive earthworms.

Moreover, in addition to plant community traits, a possible explanation for the differences in the ecosystem properties and functions across soil layers might be the different feeding strategies of different invasive earthworm species.^35^^,^^50^ Thus, earthworm community composition could have affected the soil microbial biomass and soil microbial activity,^35^ which were reduced by invasive earthworm presence and changed across soil layers. The soil microbial carbon use efficiency increased in the upper soil layer in the presence of invasive earthworms, i.e., a decrease in the soil microbial specific respiratory quotient, while the basal soil microbial activity, i.e., soil basal respiration, was lower in the invaded area with the earthworm effect being stronger in the lower soil depth. Effects of invasive earthworms on soil microbial biomass could be mediated by their impacts on soil abiotic parameters due to the burrowing and feeding activities of earthworms, which redistribute nutrients in the soil.^33^^,^^40^^,^^92^^,^^93^ Here, we showed that in the earthworm-invaded area soil pH, water, carbon, and nitrogen content declined, with a stronger reduction in the upper soil layer for water and nitrogen content, which may explain the observed decrease in soil microbial biomass and activity as also was shown by the positive correlation between these parameters (Tables S13 and S14). These results are partly in line with the results of a recent meta-analysis showing that earthworm invasion overall decreases the soil water and nitrogen content.^40^ However, these authors also observed that invasive earthworms shifted nutrient distribution between soil layers by decreasing the nutrient content in the organic soil layer while increasing that of the mineral soil layer, with this effect depending on earthworm ecological group richness.^40^ Furthermore, earthworm impacts on the soil microbial community of the rhizosphere could be mediated by changes in plant root traits, e.g., fine root length/growth^32^^,^^60^ or by changes in root exudates^94^ due to changes in plant taxonomic and functional composition. This relationship between root exudates and/or CWMs of root traits and soil microbial community in the context of invasive earthworms could also be studied in future studies to investigate the mechanism behind soil microbial community and activity changes.

### Limitations of the study

Our study suggests that invasive earthworms increase the functional similarity of the understory plant community and select for fast-growing plant species that are more efficient in nutrient uptake from the soil than slow-growing plant species. Furthermore, we show for the first time that invasive earthworms have an effect on plant functional diversity and composition, which might have cascading impacts on ecosystem functioning. However, our study represents a snapshot at a given point in time and space, which may not fully capture how the temporal and spatial dynamics of earthworm invasions affect plant communities.^85^^,^^95^ For a more comprehensive approach, and also to avoid any potential confounding effects between the physical distance between areas and the earthworm invasion status, we suggest further studies to cover larger areas in several forests by setting up several clusters of plots, widely separated, inside each of these areas, as for example performed in Fleri and Arcese.^96^ Moreover, controlled field experiments on invasive earthworm effects might be a promising approach to investigate causal relationships.^97^ We thus stress the need for standardized comparisons of invasive earthworm effects on plant (functional) diversity between multiple forests over time,^98^ to be able to gain further insights into the mechanisms that underpin the temporal dynamics of invasive earthworm effects on plant communities and ecosystem functions. Furthermore, we found that the presence of invasive earthworms drives ecosystem functions like litter decomposition and soil microbial biomass, which could be mediated by earthworm effects on plant trait diversity and composition. These causal links need to be further investigated, and adding information on belowground plant functional traits (e.g., Freschet et al.^99^) to aboveground plant traits studied could provide additional explanatory power. As some of our results may depend on earthworm community composition, we also recommend that further studies should investigate the taxonomic and functional composition of the invasive earthworm communities to better understand the mechanisms by which invasive earthworms affect plant communities and ecosystem functions.

## STAR★Methods

### Key resources table

**Table:** 

REAGENT or RESOURCE	SOURCE	IDENTIFIER
**Deposited data**		

All analyzed data and code	This study	https://doi.org/10.5281/zenodo.10245856

**Software and algorithms**		

R software version 4.3.1	R Core Team	https://www.r-project.org/

### Resource availability

#### Lead contact

Further information and requests for resources should be directed to the lead contact, Lise Thouvenot (lise.thouvenot@idiv.de).

#### Materials availability

This study did not generate new unique reagents.

#### Data and code availability

• Data have been deposited at Zenodo and are publicly available as of the date of publication. The DOI is listed in the key resources table.
• All original code has been deposited at Zenodo and is publicly available as of the date of publication. The DOI is listed in the key resources table.
• Any additional information required to reanalyze the data reported in this paper is available from the lead contact upon request.

### Method details

#### Study area and experimental design

We studied earthworm invasion effects on the understory plant community of an aspen forest located in Kananaskis Valley, in the front range of the Canadian Rocky Mountains (51°02’06’’N, 115°03’54’’W, Alberta, Canada). The diverse understory plant community is composed of grasses (*e.g. Calamagrostis rubescens*, *Leymus innovatus*), herbs and shrubs (*e.g. Aster conspicuus*, *Thalictrum venulosum, Rosa acicularis*, *Symphoricarpos occidentalis*), and two legumes species (*i.e. Lathyrus ochroleucus*, *Vicia americana*). This forest has been intensively studied for decades in the context of earthworm invasion, and the general description of the climate and soil type of the valley are detailed in previous publications^36^^,^^39^^,^^50^^,^^53^^,^^100^: the climate is considered as continental with short dry summers and cold winters (Mean annual temperature of 3.4°C and precipitation of 638 mm) and the soil as orthic grey luvisol. Notably, earthworm invasion is still ongoing in some areas of this forest, and thus parts of it are still free of earthworms. This allowed us to set up plots in non-invaded and invaded areas: twenty observation plots (1 x 1 m), representative of the understory plant community (Figure S1), were randomly distributed and established in the non-invaded and invaded areas, respectively (2 areas x 20 plots = 40 plots in total).

#### Plant diversity and traits measurements

In each of the 40 plots, we visually estimated plant species cover in June 2018, using the modified decimal scale from Londo.^101^ We used 13 cover categories (*i.e.* <1, 1-3, 3-5, 6-15, 16–25, 26–35, 36-45, 46-55, 56-65, 66–75, 76-85, 86-95, and >96%), and used their median values for further analyses. We then measured functional traits on the dominant plant species of each plot, *i.e.* plant species collectively representing at least 95% of the total cover of the plot (except for one plot where we sampled for ∼77% of the plot total cover). We measured traits related to plant growth and resource acquisition ability, as well as their responses to the environment. The height and the specific leaf area, *i.e.* the ratio of fresh leaf area to leaf dry mass, (SLA; mm^2^ mg^-1^) are correlated with plant growth rates and investment in structural tissue, while leaf dry matter content, *i.e.* the ratio of leaf dry mass to leaf fresh mass (LDMC; mg g^-1^) is positively correlated with leaf toughness and a low resource-use strategy.^102^^,^^103^^,^^104^ We also assessed leaf carbon and nitrogen content (% dry-leaf mass) that are related to plant nutritional quality and the nutrient cycling processes.^102^ To measure these traits, we selected healthy plants of dominant species in each plot or in a 5 m radius. We measured maximum plant height on at least 25 individuals, leaf traits on one to two leaves on ∼20 individuals, and foliar nutrient content on approximately 5 individuals (pool of two leaves per individual) per species and area. We note that traits were sometimes measured on fewer individuals, *e.g., Poa palustris,* depending on species abundance (Table S1). Trait values were averaged first at the individual level, and then at the species level for the invaded and non-invaded areas.

The height of the plant individuals was measured as the shortest distance between the soil and the upper leaf. To assess SLA, LDMC, and leaf nutrient content, we measured leaf lamina for grasses and either simple or compound leaves for herbs, legumes, and woody species. SLA and LDMC were determined using the partial rehydration method following the protocol of Wilson et al.^105^ Briefly, harvested leaves were wrapped in moist paper and stored in sealed plastic bags in a cooler to prevent desiccation. They were stored at 5°C overnight to promote rehydration, before being processed (within 24 h). After rehydration, fresh leaves were blotted dry and weighed with a digital balance (Sartorius CP225D). Leaf area was calculated using WinFOLIA software (Version: 2014a Pro; Regent Instruments Inc., Canada) after leaves were scanned with a flatbed scanner at 600 dpi (CanoScan Lide 220, CANON Inc, Tokyo, Japan). Leaves were oven-dried at 60°C for at least 72 h and weighed. Dry leaves were ground and 5 mg per sample were analyzed for nitrogen and carbon content using an elemental analyzer (Vario EL II, Elementar Analysensysteme GmbH, Hanau, Germany).

#### Environmental properties

We estimated the canopy openness (%) of each plot by taking pictures with a cell phone (iPhone 6+) and a clipped-on fisheye lens facing North on a tripod at a height of 1.4 m. Pictures were processed with WinSCANOPY software (Régent Instruments Inc., Québec, QC, Canada) to calculate canopy openness.

Soil abiotic conditions were assessed in half of the plots in each area, *i.e.,* 10 randomly selected plots per area, by taking two soil cores (depth 10 cm; 5 cm diameter). We measured the thickness of the different humus layers, *i.e.,* the litter (L), the organic horizons (Of and Oh), the total litter thickness, as well as the soil physicochemical properties, *i.e.* soil water content, pH, and carbon and nitrogen content. The thickness of each litter layer was averaged across soil cores for further analyses. After measuring litter thickness, each soil core was divided into two layers (0-5 cm and 5-10 cm) and then pooled per plot. Each layer of soil samples was stored separately at 5°C for shipping, after which they were sieved (2 mm mesh) and stored at -20°C. A sub-sample of sieved soil was taken, dried (60°C, for 3 days), ground and 20 mg were analyzed for carbon and nitrogen content with an elemental analyzer (Vario EL Cube, Elementar Elementar Analysensysteme GmbH, Hanau, Germany). Another subsample of approximately 2 (upper layer) or 10 g (lower layer) of sieved soil was dried at room temperature and dissolved in 12.5 or 25 ml of 0.01 M CaCl_2_, respectively, prior to measuring soil pH with a pH Meter (Orion Star A211 Benchtop) one hour later. The mass of the subsamples differed between layers, because the upper soil layer soaked most part of the CaCl_2_ solution. Thus, the ratio was adjusted by increasing the volume of CaCl_2_ solution per gram of soil for this layer: a test did not show any differences in pH when the volume changed in this range (Simone Cesarz, personal communication), and thus pH of the soil layers were still compared in the same statistical models.

To get information about the soil microbial activity, we measured basal respiration (μl O_2_ h^-1^ g^-1^ dry soil), microbial biomass (Cmic; μg C g^-1^ dry soil), and the microbial specific respiratory quotient (qO_2_; μl O_2_ μg^-1^ Cmic h^-1^) using 2.5 to 5 g of fresh soil depending on the amount of soil available, with an O_2_-microcompensation apparatus.^106^ The microbial biomass corresponds to the biomass of the whole microbial community when activated, while the basal respiration corresponds only to a part of it (*i.e.,* the active microbial community when soil samples were taken).^107^ The microbial specific respiratory quotient is negatively correlated to microbial activity (*i.e.,* microbial carbon use efficiency).^107^ Soil microbial respiration was measured every hour for 24 h at 20°C. Substrate-induced respiration was calculated after measuring the respiratory response to the addition of D-Glucose (6 mg of D-Glucose in 0.4 ml of deionized water were added per gram of fresh soil), and microbial biomass was calculated from the maximum initial respiratory response. The microbial specific respiratory quotient was calculated as the ratio of basal respiration to microbial biomass.

We also determined earthworm densities in all ten plots per area by extracting earthworms from a subplot (0.50 x 0.50 m^2^) using a combination of the hand sorting and mustard extraction methods.^36^^,^^39^ Briefly, five liters of mustard solution, consisting of 100 g of mustard powder dissolved in 10 liters of tap water and mixed with 20 ml of vinegar, were poured into the subplot after having excavated the upper ∼10 cm of soil. Topsoil was hand sorted, and earthworms were immediately collected and washed with tap water for 15 minutes. After this 15 min, the same procedure was repeated. Earthworms were stored initially in containers and then sorted into ecological groups, *i.e.*, epigeic, endogeic, and anecic, and weighed individually with a digital balance. We found three earthworm species: *Lumbricus terrestris* (anecic), *Octolasion tyrtaeum* (endogeic), and *Dendrobaena octaedra* (epigeic). In total, we extracted only one epigeic individual in the non-invaded area, which is equivalent to a density of 0.4 individuals m^-2^ and a biomass of 0.012 g m^-2^. In contrast, we extracted earthworms from three ecological groups in the invaded area, with an average density of 58 individuals m^-2^ and a biomass of 46.03 g m^-2^. Even though the uninvaded area was not completely free from earthworms, we still considered it the control area.

### Quantification and statistical analysis

All statistical analyses and figures were performed with R software version 4.3.1.^108^ To characterize the plant community, we calculated plant taxonomic diversity in terms of species richness, Shannon diversity, and Pielou’s evenness at the community and plant functional group levels using the function *specnumber* and *diversity* from the “vegan” package.^109^ These indices capture different aspects of community structure; species richness gives equal weight to common and rare species, while Shannon diversity and Pielou’s evenness give greater weight to more common species. To detect changes in dominant plant strategies, we calculated the community-weighted means (CWM)^110^ of height, SLA, LDMC, leaf carbon and nitrogen content for each plot, based on the mean trait values per species in each treatment, which we weighted with the relative median cover of plant species using the function *weighted.mean* from the “stats” package. To test whether earthworm invasion homogenizes plant communities functionally, we calculated multiple facets of functional diversity for each plot using the mean trait values per species in each treatment: standardized functional richness, functional evenness, and Rao’s entropy. We calculated these indices with the package “fundiversity”^111^ using height, SLA, LDMC, leaf carbon and nitrogen content, which were previously centered and scaled. Functional richness was standardized by the total functional richness of all sites,^111^ thus including the species from both areas. It describes the volume of the functional space of the community.^112^ Functional evenness gives information about the regularity of the distribution of the species abundance in the functional space,^78^^,^^112^ while functional entropy (Rao’s entropy) reflects the mean functional distance, *i.e.,* dissimilarity, between species weighted by their relative abundance.^113^^,^^114^

We investigated if plant community composition was dissimilar following earthworm invasion by performing a permutational multivariate analysis of variance (Permanova) on the Bray-Curtis distances between each pair of plots based on relative median plant cover using the *adonis2* function from the “vegan” package.^109^ We performed 1000 permutations, after checking for the homogeneity of group dispersion with the *betadisper* function from the “vegan” package.^109^ Both were performed with the “lingoes” correction to correct for negative eigenvalues. A Principal Coordinate Analysis (PCoA) was used to display plant community composition according to earthworm invasion status.

The effects of invasive earthworms on taxonomic plant diversity at the community level, and plant functional diversity and composition were tested using linear models of the package “stats” with Type II F-tests from the package “car”.^115^ Earthworm invasion status, *i.e.* control *vs* invaded areas, was treated as a categorical factor, and canopy openness as a continuous covariate. The same model structure was used when testing for the effect of invasive earthworms on biodiversity indices at the plant functional group level, with the difference being that the plant functional group and its interaction with earthworm invasion status were added. The effects of invasive earthworms were also tested on soil abiotic properties, *i.e.,* soil carbon and nitrogen content, soil pH, and soil water content, and soil ecosystem functions, *i.e.,* basal respiration, microbial biomass, microbial specific respiratory quotient, and litter thickness using linear models with Type II F-tests, with earthworm presence and soil depth and their interaction as factors. Soil depth was not included in the linear model when testing the effects of invasive earthworms on litter thickness. We evaluated model assumptions visually. When necessary, response variables were log-transformed (log_10_ [x +1]) to meet model assumptions: plant evenness at the community level, plant species richness, evenness, relative median cover at the plant functional group level, as well as soil carbon, nitrogen and soil microbial biomass and basal respiration. If outliers were observed, we fitted the model without them; outliers were retained when there were no changes in model estimates and the statistical significance of predictors. Similarly, we performed the analyses of the different CWM and functional diversity indices without the plot where we sampled 77% of total cover, and retain it as we did not observe changes (Table S9). Effects of invasive earthworms on the thickness of the litter layers L and Of were assessed *via* non-parametric Wilcoxon tests as model assumptions were not met.

We performed pairwise comparisons with Holm correction when model interactions were statistically significant (P < 0.05), using the package “emmeans”.^116^ Percentages of changes were calculated based on the means (for the L and Of litter thickness), and on the estimated marginal means (back-transformed when necessary) obtained with the *ggemmeans* function from the “ggeffects” package,^117^ that holds constant (averaged value) all other variables of the model. The significance codes are presented with asterisks: ∗∗∗ <0.001; ∗∗ < 0.01; ∗<0.05; (∗) <0.10. A Principal Component Analysis (PCA) was performed to visualize how CWMs (centered and scaled) varied across areas invaded by exotic earthworms using the function *prcomp* from the “stats” package. Ecosystem parameters (nutrients, microbial properties and litter) were fitted into the PCA (only correlations to axes with p-value > 0.01 were displayed), based on 20 observations (10 per area) with the *envfit* function from the "vegan" package.^109^

All figures were made with the package “ggplot2”,^118^ and all model outputs (models with and without outliers) are presented in the supplemental information. The data and code have been deposited at Zenodo: https://doi.org/10.5281/zenodo.10245856.^119^
