# Supplementary material for: Invasive earthworms can change understory plant community traits and reduce plant functional diversity
Source: iScience. 2024 Jan 29;27(3):109036. doi: 10.1016/j.isci.2024.109036 (PMC10867650; doi:10.1016/j.isci.2024.109036)
Supplement: Document S1. Figures S1 and S2 and Tables S1–S15 [file mmc1.pdf]

iScience, Volume *27*

Supplemental information

Invasive earthworms can change understory plant community traits and reduce plant functional diversity

Lise Thouvenot, Olga Ferlian, Dylan Craven, Edward A. Johnson, Johannes Köhler, Alfred Lochner, Julius Quosh, Anja Zeuner, and Nico Eisenhauer

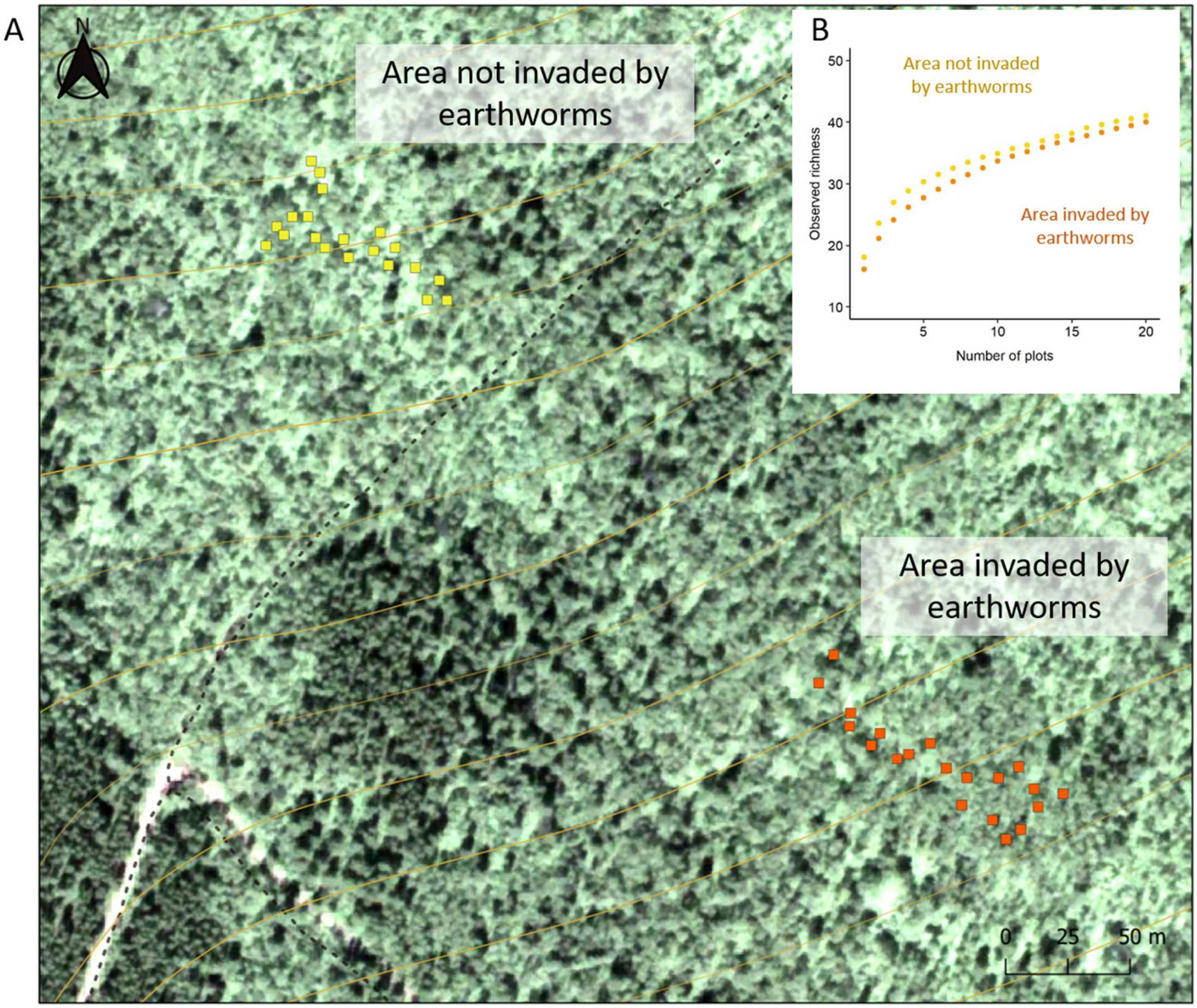

Figure S1. Map of the study site and species accumulation curves in each areas (Related to STAR Methods). A) Map of the study site located at the north of Barrier Lake, Kananaskis Valley, Alberta, Canada. (51°02' N, 115°03' W). Colors show the 20 plots in the non-invaded earthworm's area (yellow), and the 20 plots in the invaded earthworm's area (orange), distributed on the forest slope. The two areas are separated by a hiking trail (black dotted line). Mapping information: coordinate system UTM Zone 11 U, map WGS 1984, DOP data © government of Alberta 2014, DEM © University of Calgary, and mapping performed using QGIS 3.30.0 (2023). B) Species accumulation curves in each areas. Species accumulation curves were calculated using the specaccum function from the vegan package, and specifying the random method (adding sites in a random order) for 100 permutations.

Table S1. Summary table with the mean trait values for each trait and species sampled per earthworm invasion status (control *versus* invaded area) (Related to STAR Methods). Traits measured are the vegetative height (cm), the specific leaf area (SLA; mm² mg^{-1}), the leaf dry matter content (LDMC; mg g^{-1}), and the leaf carbon and nitrogen content (%). The number of individuals sampled per species is given in brackets. A slash (/) can be found when species were not sampled in this area.

Earthworm invasion status	Control area					Invaded area				
Species	Vegetative height	SLA	LDMC	leaf nitrogen content	leaf carbon content	Vegetative height	SLA	LDMC	leaf nitrogen content	leaf carbon content
Actaea rubra	47.15 (40)	29.88 (18)	238.57 (20)	2.19 (5)	46.61 (5)	/ (/)	/ (/)	/ (/)	/ (/)	/ (/)
Agoseris glauca	24.68 (25)	26.61 (22)	128.4 (22)	2.04 (5)	44.18 (5)	31.25 (24)	22.15 (24)	114 (24)	2.19 (5)	45.12 (5)
Arnica cordifolia	/ (/)	/ (/)	/ (/)	/ (/)	/ (/)	9.83 (40)	30.71 (23)	146.52 (23)	2.22 (5)	44.37 (5)
Aster conspicuus	51 (38)	24.49 (20)	229.04 (20)	2.42 (5)	46.5 (5)	42.04 (55)	28.3 (27)	199.76 (27)	2.77 (5)	46.53 (5)
Bromus ciliatus	79.91 (34)	24.9 (24)	295.31 (24)	2.75 (5)	46.86 (5)	59.16 (19)	30.07 (17)	246.24 (17)	2.68 (4)	46.6 (4)
Bromus inermis	/ (/)	/ (/)	/ (/)	/ (/)	/ (/)	63.5 (24)	25.93 (24)	259.73 (24)	2.59 (5)	46.64 (5)
Calamagrostis canadensis	57.8 (10)	37.16 (21)	341.16 (21)	2.48 (5)	45.25 (5)	57.79 (28)	24.63 (20)	363.59 (20)	2.14 (5)	41.62 (5)
Calamagrostis rubescens	33.9 (41)	25.6 (23)	420.94 (23)	2.04 (5)	44.24 (5)	32.43 (75)	28.23 (50)	350.25 (50)	2.39 (5)	43.39 (5)
Carex siccata	/ (/)	/ (/)	/ (/)	/ (/)	/ (/)	31.28 (25)	26.96 (20)	342.6 (20)	1.7 (5)	43.56 (5)
Castilleja miniata	40.2 (30)	29.22 (23)	222.05 (23)	3.67 (5)	42.13 (5)	/ (/)	/ (/)	/ (/)	/ (/)	/ (/)
Chamerion angustifolium	45.5 (40)	32.12 (20)	265.8 (20)	2.59 (5)	43.8 (5)	25.18 (39)	32.34 (29)	208.45 (29)	2.64 (5)	45.64 (5)
Clematis occidentalis	50.63 (27)	26.68 (22)	217.22 (22)	2.48 (5)	44.85 (5)	31.39 (31)	32.91 (26)	185.45 (26)	2.29 (5)	44.4 (5)
Delphinium glaucum	75.03 (34)	28.66 (20)	186.06 (20)	3.15 (5)	44.19 (5)	63.97 (33)	29.84 (21)	169.75 (21)	3.46 (5)	44.7 (5)
Elymus trachycaulus	58.08 (26)	35.67 (20)	285.74 (20)	3.18 (5)	43.75 (5)	/ (/)	/ (/)	/ (/)	/ (/)	/ (/)
Fragaria virginiana	18.86 (43)	20.05 (22)	244.7 (22)	1.89 (5)	44.06 (5)	15.89 (44)	21.63 (25)	247.32 (25)	1.55 (5)	44.01 (5)
Galium boreale	36.73 (40)	30.15 (20)	271.12 (20)	2.5 (5)	44.84 (5)	40.44 (48)	30.41 (26)	229.83 (26)	2.63 (5)	44.61 (5)
Geranium richardsonii	33.53 (43)	21.13 (19)	177.93 (20)	2.47 (5)	45.71 (5)	26.57 (49)	24.65 (24)	172.57 (24)	2.49 (5)	46.28 (5)
Heracleum lanatum	47.29 (42)	19.92 (15)	148.95 (20)	2.83 (5)	43.22 (5)	31.3 (47)	26.46 (20)	141.7 (22)	3.25 (5)	42.95 (5)
Lathyrus ochroleucus	37.5 (40)	38.92 (20)	226.67 (20)	4.17 (5)	45.2 (5)	32.13 (56)	43.09 (27)	192.79 (27)	5.26 (5)	45.52 (5)
Leymus innovatus	33.53 (30)	25.17 (25)	284.22 (25)	2.33 (5)	43.44 (5)	31.08 (72)	26.49 (51)	284.35 (52)	2.32 (5)	43.03 (5)
Lilium philadelphicum	48.72 (25)	26.32 (20)	157.36 (20)	3.12 (5)	43.85 (5)	39.84 (44)	31.46 (28)	135.05 (28)	2.93 (5)	44.28 (5)
Maianthemum stellatum	25.12 (33)	27.89 (21)	179.08 (21)	2.01 (5)	42.95 (5)	/ (/)	/ (/)	/ (/)	/ (/)	/ (/)
Osmorhiza depauperata	19.5 (44)	36.68 (27)	171.88 (27)	1.59 (5)	40.01 (5)	14.06 (48)	37.9 (36)	162.87 (36)	2.03 (5)	41.46 (5)
Packera paupercula	/ (/)	/ (/)	/ (/)	/ (/)	/ (/)	9.5 (26)	29.1 (20)	138.59 (20)	1.88 (5)	44.87 (5)
Pedicularis bracteosa	/ (/)	/ (/)	/ (/)	/ (/)	/ (/)	34.67 (24)	24.45 (23)	221.38 (24)	2.66 (5)	39.32 (5)
Poa palustris	26 (6)	43.31 (23)	254.49 (23)	1.74 (4)	31.46 (4)	/ (/)	/ (/)	/ (/)	/ (/)	/ (/)
Populus balsamifera	109.88 (33)	14.18 (23)	369.6 (23)	2.05 (5)	46.31 (5)	/ (/)	/ (/)	/ (/)	/ (/)	/ (/)
Populus tremuloides	94.83 (41)	15.16 (21)	372.61 (21)	2.39 (5)	49.03 (5)	65.75 (48)	18.52 (24)	297.41 (24)	3.04 (5)	48.24 (5)
Prosartes trachycarpa	30.15 (40)	38.57 (20)	202.11 (20)	2.64 (5)	44.59 (5)	20.54 (46)	38.86 (27)	189.69 (27)	3.05 (5)	44.63 (5)
Prunus virginiana	79.45 (33)	19.3 (24)	346.24 (24)	2.26 (5)	45.52 (5)	/ (/)	/ (/)	/ (/)	/ (/)	/ (/)
Pyrola asarifolia	/ (/)	/ (/)	/ (/)	/ (/)	/ (/)	4.97 (32)	18.7 (21)	288.3 (21)	1.77 (5)	47.94 (5)
Rosa acicularis	44.21 (42)	23.55 (20)	358.17 (21)	2.13 (5)	47.13 (5)	29.09 (45)	25.96 (22)	323.14 (22)	2.05 (5)	45.61 (5)
Rubus idaeus	37.74 (39)	29.9 (23)	304.12 (23)	2.15 (5)	45.19 (5)	26.97 (30)	29.79 (20)	299 (21)	2.34 (5)	47.04 (5)
Rubus pubescens	/ (/)	/ (/)	/ (/)	/ (/)	/ (/)	14.47 (34)	39.63 (21)	259.74 (21)	1.75 (5)	44.35 (5)
Sanicula marilandica	/ (/)	/ (/)	/ (/)	/ (/)	/ (/)	26.91 (32)	26.28 (21)	172.33 (21)	2.18 (5)	43.64 (5)
Spiraea betulifolia	30.62 (37)	30.04 (21)	410.15 (21)	2.16 (5)	45.3 (5)	23.77 (26)	27.74 (21)	408.18 (21)	1.75 (5)	45.44 (5)
Symphoricarpos albus	36.55 (38)	20.85 (23)	425.86 (23)	1.9 (5)	47.1 (5)	39.64 (33)	22.07 (20)	401.88 (20)	1.99 (5)	47.05 (5)
Symphoricarpos occidentalis	63.16 (38)	16.73 (21)	371.47 (21)	1.6 (5)	47.44 (5)	/ (/)	/ (/)	/ (/)	/ (/)	/ (/)
Symphyotrichum ciliolatum	18.1 (40)	27.72 (20)	168.88 (20)	1.96 (5)	41.74 (5)	15.67 (56)	30.98 (27)	159.44 (27)	2.3 (5)	41.77 (5)
Taraxacum officinale	/ (/)	/ (/)	/ (/)	/ (/)	/ (/)	23.46 (28)	36.38 (23)	130.35 (23)	1.76 (5)	41.26 (5)
Thalictrum venulosum	57.63 (40)	35.73 (20)	297.31 (20)	3.35 (5)	45.62 (5)	29.02 (42)	35.76 (23)	267.04 (23)	3.17 (5)	45.43 (5)
Vicia americana	46 (40)	43.78 (20)	200.21 (20)	4.41 (5)	45.11 (5)	31.15 (46)	45.83 (28)	195.02 (28)	4.98 (5)	45.67 (5)
Viola canadensis	18.53 (40)	35.07 (20)	162.43 (20)	2.7 (5)	42.21 (5)	17.95 (42)	34.47 (20)	172.28 (20)	3.16 (5)	42.3 (5)

Table S2. Effects of invasive earthworms on plant taxonomic diversity (Related to Figure 1). Results of the model on the effects of invasive earthworms (*i.e.* "Earthworm invasion status (Invaded)") and the canopy openness on the total richness of the plant community, its Shannon diversity, and the evenness of the plant community (log10 (X+1)). Data were analyzed using linear models with earthworm invasion status and canopy openness as explanatory variables. The transformation applied to the data, the number of observations as well as the R^2 are also presented.

	**Species richness**			**Shannon diversity**			**Log 10 (Evenness+1)**		
Predictors	*Estimates (Standard error)*	*t value*	*p-value*	*Estimates (Standard error)*	*t value*	*p-value*	*Estimates (Standard error)*	*t value*	*p-value*
Intercept	15.01 (2.91)	5.15	**<0.001**	2.45 (0.30)	8.31	**<0.001**	0.28 (0.02)	15.09	**<0.001**
Earthworm invasion status (Invaded)	-1.29 (0.84)	- 1.53	0.134	-0.16 (0.09)	- 1.88	0.068	-0.01 (0.01)	-1.55	0.129
Canopy openness	0.06 (0.06)	0.94	0.354	0.00 (0.01)	0.12	0.907	-0.00 (0.00)	-0.43	0.673
Observations	40			40			40		
R^2 / R^2 adjusted	0.150 / 0.104			0.123 / 0.076			0.066 / 0.016		

Table S3. Effects of invasive earthworms on plant functional group diversity and cover (Related to Figure 2). Results of the model on the effects of invasive earthworms (*i.e.* "Earthworm invasion status (Invaded)") alone and in interaction with the plant functional group as well as the canopy openness as a covariate on the richness (log10 (X+1)), Shannon diversity, evenness (log10 (X+1)) and relative median cover (log10 (X+1)) of the plant functional groups. Data were analyzed using linear models with earthworm invasion status and functional group as factors and canopy openness as a covariate. The transformation applied to the data, the number of observations, as well as the R^2, are also presented.

	**log 10(Richness+1)**			**Shannon diversity**			**log 10(Eveness+1)**			**log 10(Relative median cover+1)**		
Predictors	*Estimates (Standard error)*	*t value*	*p-value*	*Estimates (Standard error)*	*t value*	*p-value*	*Estimates (Standard error)*	*t value*	*p-value*	*Estimates (Standard error)*	*t value*	*p-value*
(Intercept)	0.50 (0.07)	6.95	**<0.001**	0.55 (0.22)	2.56	**0.011**	0.24 (0.03)	8.00	**<0.001**	1.46 (0.14)	10.25	**<0.001**
Functional group (Herb)	0.49 (0.03)	14.36	**<0.001**	1.25 (0.10)	12.29	**<0.001**	0.03 (0.01)	1.96	0.052	0.54 (0.07)	8.01	**<0.001**
Functional group (Legume)	-0.13 (0.03)	-3.69	**<0.001**	-0.20 (0.10)	-1.94	0.054	0.03 (0.01)	1.86	0.065	-0.40 (0.07)	-5.95	**<0.001**
Functional group (Woody)	0.08 (0.03)	2.44	**0.016**	0.27 (0.10)	2.63	**0.009**	0.02 (0.01)	1.15	0.252	0.10 (0.07)	1.50	0.136
Earthworm invasion status (Invaded)	0.01 (0.04)	0.20	0.840	0.07 (0.11)	0.66	0.511	0.01 (0.01)	1.01	0.315	0.27 (0.07)	3.82	**<0.001**
Canopy openness	0.00 (0.00)	0.58	0.564	0.00 (0.00)	0.53	0.600	-0.00 (0.00)	-0.05	0.961	-0.01 (0.00)	-1.85	0.067
Functional group (Herb) * Earthworm invasion status (Invaded)	-0.01 (0.05)	-0.16	0.870	-0.05 (0.14)	-0.33	0.744	-0.01 (0.02)	-0.63	0.531	-0.35 (0.09)	-3.72	**<0.001**
Functional group (Legume) * Earthworm invasion status (Invaded)	0.02 (0.05)	0.47	0.639	-0.10 (0.14)	-0.70	0.483	-0.04 (0.02)	-1.97	0.051	-0.28 (0.09)	-2.97	**0.003**
Functional group (Woody) * Earthworm invasion status (Invaded)	-0.21 (0.05)	-4.44	**<0.001**	-0.55 (0.14)	-3.77	**<0.001**	0.00 (0.02)	0.07	0.943	-0.76 (0.09)	-8.07	**<0.001**
Observations	160			158			138			160		
R^2 / R^2 adjusted	0.840 / 0.832			0.782 / 0.770			0.073 / 0.016			0.757 / 0.744		

Table S4. Effects of invasive earthworms on plant functional diversity (Related to Figure 3). Results of the model on the effects of invasive earthworms (*i.e.* "Earthworm invasion status (Invaded)") and the canopy openness on the standardized functional richness, functional evenness, and Rao's entropy index of the plant community. Data were analyzed using linear models with earthworm invasion status and canopy openness as explanatory variables. The number of observations and R^2 are also presented.

	**Functional richness**			**Functional evenness**			**Rao's Q**		
Predictors	*Estimates (Standard error)*	*t value*	*p-value*	*Estimates (Standard error)*	*t value*	*p-value*	*Estimates (Standard error)*	*t value*	*p-value*
Intercept	0.04 (0.03)	1.59	0.121	0.79 (0.08)	10.14	**<0.001**	2.39 (0.30)	7.96	**<0.001**
Earthworm invasion status (Invaded)	-0.02 (0.01)	-2.75	**0.009**	-0.09 (0.02)	-3.84	**<0.001**	-0.28 (0.09)	-3.21	**0.003**
Canopy openness	0.00 (0.00)	0.66	0.514	-0.00 (0.00)	-0.81	0.424	0.00 (0.01)	0.07	0.943
Observations	40			40			40		
R^2 / R^2 adjusted	0.269 / 0.229			0.311 / 0.274			0.281 / 0.242		

Table S5. Effects of invasive earthworms on plant community traits (Related to Figure 4).
Results of the model on the effects of invasive earthworms (*i.e.* "Earthworm invasion status (Invaded)") and the canopy openness on the community-weighted means of the height, specific leaf area (SLA), leaf dry matter content (LDMC), leaf carbon and leaf nitrogen content of the plant community. Data were analyzed using linear models with earthworm invasion status and canopy openness as explanatory variables. The number of observations and R^2 are also presented.

	**CWM height**			**CWM SLA**			**CWM LDMC**			**CWM leaf carbon**			**CWM leaf nitrogen**		
Predictors	*Estimates (Standard error)*	*t value*	*p-value*	*Estimates (Standard error)*	*t value*	*p-value*	*Estimates (Standard error)*	*t value*	*p-value*	*Estimates (Standard error)*	*t value*	*p-value*	*Estimates (Standard error)*	*t value*	*p-value*
Intercept	34.50 (4.83)	7.15	**<0.001**	29.97 (1.20)	24.88	**<0.001**	270.33 (25.21)	10.72	**<0.001**	45.63 (0.57)	80.68	**<0.001**	2.56 (0.16)	16.52	**<0.001**
Earthworm invasion status (Invaded)	-12.80 (1.39)	-9.20	**<0.001**	2.75 (0.35)	7.92	**<0.001**	-21.77 (7.27)	-2.99	**0.005**	-1.04 (0.16)	-6.36	**<0.001**	0.22 (0.04)	4.99	**<0.001**
Canopy openness	0.23 (0.11)	2.19	**0.035**	-0.08 (0.03)	-2.96	**0.005**	-0.16 (0.55)	-0.28	0.778	-0.01 (0.01)	-0.86	0.397	-0.00 (0.00)	-0.48	0.637
Observations	40			40			40			40			40		
R^2 / R^2 adjusted	0.804 / 0.794			0.781 / 0.769			0.233 / 0.191			0.569 / 0.546			0.507 / 0.480		

Table S6. Effects of invasive earthworms on soil abiotic and biotic parameters (Related to Figure 6 and Figure 8). Results of the model on the effects of invasive earthworms (*i.e.* "Earthworm invasion status (Invaded)") and soil depth (i.e. " Soil depth (5 -10 cm)") on the soil pH, soil carbon (soil C), nitrogen (soil N) and water (H_2O) content as well as the microbial biomass (Cmic) and activity (i.e. basal respiration and qO_2). Data were analyzed using linear models with earthworm invasion status and soil depth alone and in interaction as explanatory variables. The transformation applied to the data, the number of observations, as well as the R^2, are also presented.

	**soil pH**			**log 10(soil C+1)**			**log 10(soil N+1)**			**H_2O**			**log 10(Cmic+1)**			**log 10(basal respiration+1)**			**qO_2**		
Predictors	*Estimates (Standard error)*	*t value*	*p-value*	*Estimates (Standard error)*	*t value*	*p-value*	*Estimates (Standard error)*	*t value*	*p-value*	*Estimates (Standard error)*	*t value*	*p-value*	*Estimates (Standard error)*	*t value*	*p-value*	*Estimates (Standard error)*	*t value*	*p-value*	*Estimates (Standard error)*	*t value*	*p-value*
(Intercept)	6.28 (0.10)	60.24	**<0.001**	1.51 (0.06)	23.91	**<0.001**	0.43 (0.02)	18.14	**<0.001**	55.56 (2.62)	21.24	**<0.001**	3.74 (0.07)	50.16	**<0.001**	1.47 (0.06)	22.75	**<0.001**	0.01 (0.00)	16.33	**<0.001**
Earthworm invasion status (Invaded)	-0.13 (0.15)	-0.89	0.380	-0.37 (0.09)	-4.18	**<0.001**	-0.21 (0.03)	-6.27	**<0.001**	-17.21 (3.70)	-4.65	**<0.001**	-0.40 (0.11)	-3.75	**0.001**	-0.52 (0.09)	-5.67	**<0.001**	-0.00 (0.00)	-3.55	**0.001**
Soil depth (5 -10cm)	-0.35 (0.15)	-2.38	**0.023**	-0.61 (0.09)	-6.88	**<0.001**	-0.27 (0.03)	-7.96	**<0.001**	-25.13 (3.70)	-6.79	**<0.001**	-0.83 (0.11)	-7.88	**<0.001**	-0.80 (0.09)	-8.70	**<0.001**	-0.00 (0.00)	-1.42	0.164
Earthworms area (Invaded): Soil depth (5 -10cm)	-0.18 (0.21)	-0.85	0.399	0.13 (0.13)	1.02	0.316	0.14 (0.05)	3.07	**0.004**	10.97 (5.23)	2.10	**0.043**	0.15 (0.15)	1.02	0.315	0.32 (0.13)	2.46	**0.019**	0.00 (0.00)	2.54	**0.016**
Observations	40			40			40			40			40			39			39		
R^2 / R^2 adjusted	0.390 / 0.339			0.737 / 0.715			0.754 / 0.733			0.692 / 0.666			0.772 / 0.753			0.786 / 0.768			0.270 / 0.207		

Table S7. Effects of invasive earthworms on litter layers (Related to Figure 7). Results of the model on the effects of invasive earthworms (*i.e.* "Earthworm invasion status (Invaded)") on the total humus layer and Oh layer. Data were analyzed using linear models with earthworm invasion status as explanatory variable. The number of observations, as well as the R^2, are also presented.

	**Thickness of the total litter layer**			**Thickness of the Oh layer**		
Predictors	*Estimates (Standard error)*	*t value*	*p-value*	*Estimates (Standard error)*	*t value*	*p-value*
(Intercept)	7.25 (0.71)	10.16	**<0.001**	4.05 (0.54)	7.47	**<0.001**
Earthworm invasion status (Invaded)	-2.05 (1.01)	-2.03	0.057	0.40 (0.77)	0.52	0.608
Observations	20			20		
R^2 / R^2 adjusted	0.186 / 0.141			0.015 / -0.040		

Table S8. Results of the linear model without outliers on the effects of invasive earthworms and functional group and/or canopy openness or soil depth on the evenness of the plant community, the relative cover, richness, and evenness of plant functional groups, and the soil carbon and nitrogen contents (Related to STAR Methods, Figure 1, Figure 2 and Figure 6). The transformation applied to the data, the outlier(s) removed, the number of observations, as well as the R^2, are also presented.

	**log 10(community evenness+1)**			**log 10(functional group relative median cover+1)**			**log 10(functional group richness+1)**			**log 10(functional group eveness+1)**			**log 10(soil C+1)**			**log 10(soil N+1)**		
Predictors	*Estimates (Standard error)*	*t value*	*p-value*	*Estimates (Standard error)*	*t value*	*p-value*	*Estimates (Standard error)*	*t value*	*p-value*	*Estimates (Standard error)*	*t value*	*p-value*	*Estimates (Standard error)*	*t value*	*p-value*	*Estimates (Standard error)*	*t value*	*p-value*
(Intercept)	0.28 (0.01)	20.59	**<0.001**	1.43 (0.13)	11.20	**<0.001**	0.49 (0.07)	7.52	**<0.001**	0.24 (0.03)	9.64	**<0.001**	1.59 (0.05)	29.38	**<0.001**	0.46 (0.02)	25.91	**<0.001**
Earthworm invasion status [Invaded]	-0.01 (0.00)	-1.39	0.172	0.27 (0.06)	4.34	**<0.001**	0.01 (0.03)	0.30	0.765	0.01 (0.01)	1.16	0.247	-0.45 (0.07)	-6.04	**<0.001**	-0.24 (0.02)	-9.88	**<0.001**
Canopy openness	-0.00 (0.00)	-0.73	0.471	-0.00 (0.00)	-1.81	0.072	0.00 (0.00)	0.90	0.370	-0.00 (0.00)	-0.03	0.978	/	/	/	/	/	/
functional group [Herb]	/	/	/	0.54 (0.06)	8.95	**<0.001**	0.49 (0.03)	16.03	**<0.001**	0.02 (0.01)	1.63	0.106	/	/	/	/	/	/
functional group [Legume]	/	/	/	-0.36 (0.06)	-5.87	**<0.001**	-0.10 (0.03)	-3.35	**0.001**	0.02 (0.01)	1.56	0.120	/	/	/	/	/	/
functional group [Woody]	/	/	/	0.10 (0.06)	1.67	0.096	0.08 (0.03)	2.72	**0.007**	0.01 (0.01)	1.23	0.223	/	/	/	/	/	/
functional group [Herb] * Earthworm invasion status [Invaded]	/	/	/	-0.35 (0.08)	-4.15	**<0.001**	-0.01 (0.04)	-0.18	0.854	-0.01 (0.02)	-0.73	0.468	/	/	/	/	/	/
functional group [Legume] * Earthworm invasion status [Invaded]	/	/	/	-0.32 (0.09)	-3.79	**<0.001**	0.00 (0.04)	0.01	0.990	-0.03 (0.02)	-1.78	0.077	/	/	/	/	/	/
functional group [Woody] * Earthworm invasion status [Invaded]	/	/	/	-0.72 (0.09)	-8.44	**<0.001**	-0.19 (0.04)	-4.42	**<0.001**	-0.00 (0.02)	-0.27	0.789	/	/	/	/	/	/
Soil depth (5 -10cm)	/	/	/	/	/	/	/	/	/	/	/	/	-0.69 (0.07)	-9.28	**<0.001**	-0.30 (0.02)	-12.19	**<0.001**
Earthworm invasion status (Invaded) : Soil depth (5 -10cm)	/	/	/	/	/	/	/	/	/	/	/	/	0.20 (0.10)	1.97	0.057	0.18 (0.03)	5.21	**<0.001**
Observations (Removed outliers)	39 (FO2018_P22, evenness value of 0.607)			158 (FO2018_P04, Legume, richness value of 0; FO2018_P39, Woody, richness value of 0)			158 (FO2018_P04, Legume, richness value of 0; FO2018_P39, Woody, richness value of 0)			134 (FO2018_P20, Grass, evenness value of 0.276; FO2018_P22, Grass, evenness value of 0.303; FO2018_P27, Legume, evenness value of 0.276; FO2018_P08, Woody, evenness value of 0.368)			39 (FO2018_P12, depth 0-5cm, soil carbon value of 5.716)			39 (FO2018_P12, depth 0-5cm, soil nitrogen value of 0.339)		
R^2 / R^2 adjusted	0.051 / -0.002			0.783 / 0.772			0.864 / 0.856			0.052 / -0.009			0.831 / 0.816			0.876 / 0.865		

Table S9. Results of the linear model without plot 20 (where we sampled ~77% of the total cover of the plot) on the effects of invasive earthworms and canopy openness on the CWM and functional diversity indices (Related to STAR Methods, Figure 3 and Figure 4). The number of observations as well as the R^2 are also presented.

	**CWM height**			**CWM SLA**			**CWM LDMC**			**CWM leaf carbon**			**CWM leaf nitrogen**			**Functional richness**			**Functional evenness**			**Rao's Q**		
Predictors	*Estimates (Standard error)*	*t value*	*p-value*	*Estimates (Standard error)*	*t value*	*p-value*	*Estimates (Standard error)*	*t value*	*p-value*	*Estimates (Standard error)*	*t value*	*p-value*	*Estimates (Standard error)*	*t value*	*p-value*	*Estimates (Standard error)*	*t value*	*p-value*	*Estimates (Standard error)*	*t value*	*p-value*	*Estimates (Standard error)*	*t value*	*p-value*
Intercept	35.57 (4.81)	7.40	**<0.001**	29.82 (1.22)	24.35	**<0.001**	275.73 (25.18)	10.95	**<0.001**	45.76 (0.56)	81.22	**<0.001**	2.54 (0.16)	16.20	**<0.001**	0.04 (0.03)	1.49	0.146	0.80 (0.08)	10.21	**<0.001**	2.48 (0.29)	8.52	**<0.001**
Earthworm invasion status (Invaded)	-13.20 (1.40)	-9.44	**<0.001**	2.81 (0.36)	7.88	**<0.001**	-23.79 (7.32)	-3.25	**0.003**	-1.08 (0.16)	-6.62	**<0.001**	0.23 (0.05)	5.12	**<0.001**	-0.02 (0.01)	-2.58	**0.014**	-0.09 (0.02)	-3.98	**<0.001**	-0.31 (0.08)	-3.68	**0.001**
Canopy openness	0.21 (0.11)	2.04	**0.049**	-0.08 (0.03)	-2.83	**0.008**	-0.25 (0.55)	-0.45	0.657	-0.01 (0.01)	-1.04	0.307	-0.00 (0.00)	-0.35	0.729	0.00 (0.00)	0.70	0.489	-0.00 (0.00)	-0.93	0.358	-0.00 (0.01)	-0.16	0.871
Observations	39			39			39			39			39			39			39			39		
R^2 / R^2 adjusted	0.815 / 0.805			0.785 / 0.773			0.263 / 0.222			0.593 / 0.571			0.522 / 0.496			0.260 / 0.218			0.331 / 0.294			0.334 / 0.297		

Table S10: Contributions of CWMs of different plant traits to each axis of the PCA (Related to Figure 5).

	Dim.1	Dim.2
CWM Height	22.94011	10.10897
CWM SLA	24.52348	2.181019
CWM LDMC	12.51091	59.92852
CWM Carbon	20.29624	17.69152
CWM Nitrogen	19.72927	10.08997

Table S11: Effects of invasive earthworms on ecosystem functions (Related to Figure 5). Ecosystem functions fitted into the PCA, with the envfit function from the vegan package. R^2 gives the correlation coefficient/ goodness of fit for each ecosystem functions. The p-values give the significance for each ecosystem function after 1000 permutations. The PCA 1 and PC2 give the coordinates of the head, and the direction for each ecosystem functions (scaled).

Ecosystem functions	R^2	P-values	PC1	PC2
soil nitrogen, depth 0-5	0.597	**0.004**	-0.691	0.346
soil nitrogen depth 5-10	0.279	*0.074*	-0.526	-0.053
soil carbon depth 0-5	0.569	**0.003**	-0.719	0.227
soil carbon depth 5-10	0.217	0.134	-0.466	-0.009
soil pH, depth 0-5	0.181	0.212	-0.045	0.423
soil pH, depth 5-10	0.249	*0.096*	-0.497	-0.043
Microbial basal respiration, depth 0-5	0.648	**0.001**	-0.789	0.158
Microbial basal respiration, depth 5-10	0.333	**0.039**	-0.576	-0.034
Cmic, depth 0-5	0.623	**0.002**	-0.762	0.206
Cmic, depth 5-10	0.222	0.138	-0.47	-0.033
microbial specific respiratory quotient, depth 0-5	0.515	**0.004**	-0.716	0.035
microbial specific respiratory quotient, depth 5-10	0.003	0.975	0.023	0.054
Water content, depth 0-5	0.422	**0.016**	-0.629	0.164
Water content, depth 5-10	0.362	**0.037**	-0.505	0.327
Humus layer thickness	0.175	0.218	-0.309	-0.283

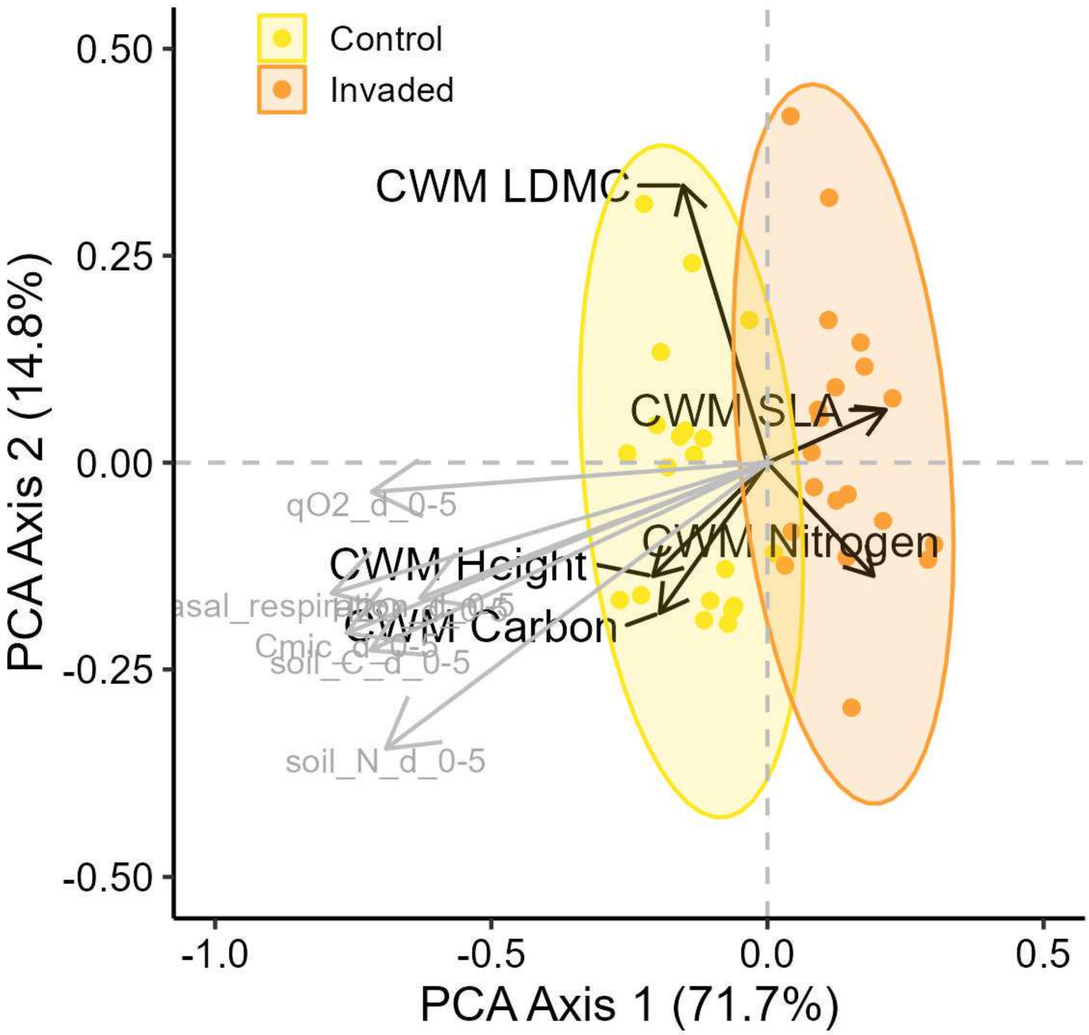

Figure S2: Visualization of soil abiotic properties and ecosystem functions according to earthworm invasion areas (Related to Figure 5). Principal component analysis (PCA) with the five CWMs of plant traits to characterize the earthworm invasion areas, and the ecosystem functions with a p-value < 0.01 displayed only, plotted with the "envfit" function from the vegan package (Oksanen et al., 2019). CWMs were centered and scaled, and percentage of variance explain for each axis in in brackets. CWMs are plotted based on 40 observations in total (20 per areas), while ecosystem functions are plotted based on 20 observations (10 per areas) as they were measured in half of the plots. Contributions of CWM to each axis can be found in Table S10, and the fit of ecosystem functions to axes in Table S11.

Table S12: Spearman's correlations between functional diversity and community weighted means of the plant community in the control and invaded area (Related to Figure 3, Figure 4 and Discussion). Correlation coefficients and p-values (in brackets) above the diagonal correspond to the ones in the control area, while those below the diagonal are those from the invaded area. Abbreviations are: SLA=specific leaf area; LDMC=leaf dry matter content. Number of observations per area: 20. Significant correlations are highlighted in bold, tendencies are in italic.

		Control area							
		FRic	FEve	Q	CWM Height	CWM SLA	CWM LDMC	CWM Carbon	CWM Nitrogen
Invaded area	FRic		0.229 (0.332)	**0.484 (0.031)**	0.226 (0.339)	0.259 (0.271)	0.095 (0.691)	-0.104 (0.663)	*0.386 (0.092)*
	FEve	-0.057 (0.811)		0.263 (0.262)	-0.098 (0.682)	0.304 (0.193)	0.232 (0.326)	0.066 (0.782)	0.092 (0.701)
	Q	**0.504 (0.024)**	0.144 (0.544)		*0.403 (0.078)*	-0.129 (0.587)	0.182 (0.443)	0.343 (0.139)	0.051 (0.83)
	CWM Height	0.017 (0.945)	-0.03 (0.9)	0.107 (0.654)		**-0.474 (0.035)**	0.102 (0.668)	**0.678 (0.001)**	0.005 (0.985)
	CWM SLA	**-0.632 (0.003)**	0.014 (0.955)	-0.095 (0.691)	*-0.391 (0.088)*		-0.167 (0.482)	-0.256 (0.277)	**0.504 (0.024)**
	CWM LDMC	-0.301 (0.198)	*0.421 (0.064)*	-0.122 (0.609)	0.338 (0.145)	-0.14 (0.556)		0.25 (0.289)	**-0.451 (0.046)**
	CWM Carbon	0.353 (0.126)	-0.159 (0.502)	*0.427 (0.06)*	0.343 (0.139)	**-0.46 (0.041)**	-0.026 (0.915)		-0.146 (0.539)
	CWM Nitrogen	*-0.438 (0.054)*	-0.029 (0.905)	0.177 (0.454)	0.02 (0.935)	**0.606 (0.005)**	*-0.391 (0.088)*	-0.114 (0.631)	

Table S13: Spearman's correlation coefficients and p-values (in brackets) between community weighted means of the plant community and the total litter thickness in the control and invaded areas (Related to Figure 4, Figure 7 and Discussion). Abbreviations are: SLA=specific leaf area; LDMC=leaf dry matter content; Number of observations per area: 10. Significant correlations are highlighted in bold.

		CWM Height	CWM SLA	CWM LDMC	CWM Carbon	CWM Nitrogen
Litter thickness	Control area	0.044 (0.904)	-0.15 (0.679)	0.532 (0.114)	0.006 (0.986)	**-0.838 (0.002)**
	Invaded area	-0.492 (0.148)	-0.255 (0.476)	-0.213 (0.555)	-0.17 (0.638)	-0.377 (0.283)

Table S14: Spearman's correlations between community weighted means of the plant community and the soil abiotic and biotic parameters in the upper soil layer (0 to 5 cm) in the control and invaded area (Related to Figure 6, Figure 8 and Discussion). Correlation coefficients and p-values (in brackets) above the diagonal correspond to the ones in the control area, while those below the diagonal are those from the invaded area. Abbreviations are: qO_2 = microbial specific respiratory quotient; BR = Basal respiration; SLA=specific leaf area; LDMC=leaf dry matter content. Correlations between CWMs of plant traits are not shown, as correlation were not performed with the full dataset (see Table S9). Number of observations per area: 9 to 10. Significant correlations (P < 0.05) are highlighted in bold, trends (P < 0.1) are given in italic.

Soil depth 0 - 5 cm		Control area											
		Soil N	Soil C	Soil pH	BR	Cmic	qO_2	H_2O	CWM Height	CWM SLA	CWM LDMC	CWM Carbon	CWM Nitrogen
Invaded area	Soil N		**0.806 (0.005)**	0.188 (0.603)	0.491 (0.15)	**0.879 (0.001)**	0.212 (0.556)	**0.806 (0.005)**	0.297 (0.405)	-0.006 (0.987)	-0.176 (0.627)	0.345 (0.328)	-0.115 (0.751)
	Soil C	**0.964 (0)**		0.212 (0.556)	**0.879 (0.001)**	**0.709 (0.022)**	**0.697 (0.025)**	**0.782 (0.008)**	0.345 (0.328)	-0.152 (0.676)	-0.006 (0.987)	0.055 (0.881)	-0.455 (0.187)
	Soil pH	0.491 (0.15)	0.309 (0.385)		0.333 (0.347)	0.333 (0.347)	0.079 (0.829)	-0.067 (0.855)	-0.176 (0.627)	0.236 (0.511)	-0.103 (0.777)	-0.297 (0.405)	0.103 (0.777)
	BR	**0.855 (0.002)**	**0.867 (0.001)**	0.285 (0.425)		0.539 (0.108)	**0.891 (0.001)**	*0.564 (0.09)*	0.358 (0.31)	-0.127 (0.726)	0.285 (0.425)	0.006 (0.987)	-0.527 (0.117)
	Cmic	**0.782 (0.008)**	**0.818 (0.004)**	0.261 (0.467)	**0.952 (0)**		0.2 (0.58)	*0.588 (0.074)*	0.515 (0.128)	-0.2 (0.58)	0.188 (0.603)	0.442 (0.2)	-0.091 (0.803)
	qO_2	**0.661 (0.038)**	*0.612 (0.06)*	0.115 (0.751)	**0.758 (0.011)**	**0.673 (0.033)**		0.479 (0.162)	0.248 (0.489)	-0.018 (0.96)	0.358 (0.31)	-0.018 (0.96)	**-0.636 (0.048)**
	H_2O	**0.867 (0.001)**	**0.927 (0)**	0.236 (0.511)	**0.867 (0.001)**	**0.794 (0.006)**	**0.588 (0.074)**		0.188 (0.603)	0.261 (0.467)	-0.152 (0.676)	0.394 (0.26)	-0.321 (0.365)
	CWM Height	-0.37 (0.293)	-0.321 (0.365)	-0.285 (0.425)	-0.091 (0.803)	0.067 (0.855)	0.067 (0.855)	-0.394 (0.26)		/	/	/	/
	CWM SLA	-0.115 (0.751)	-0.152 (0.676)	-0.321 (0.365)	-0.091 (0.803)	-0.152 (0.676)	-0.006 (0.987)	-0.164 (0.651)	/		/	/	/
	CWM LDMC	-0.479 (0.162)	-0.297 (0.405)	**-0.709 (0.022)**	-0.176 (0.627)	-0.115 (0.751)	-0.115 (0.751)	-0.236 (0.511)	/	/		/	/
	CWM Carbon	0.127 (0.726)	-0.055 (0.881)	*0.564 (0.09)*	0.067 (0.855)	0.079 (0.829)	0.273 (0.446)	-0.2 (0.58)	/	/	/		/
	CWM Nitrogen	-0.152 (0.676)	-0.212 (0.556)	-0.139 (0.701)	-0.139 (0.701)	-0.079 (0.829)	-0.042 (0.907)	-0.285 (0.425)	/	/	/	/	

Table S15: Spearman's correlations between community weighted means of the plant community and the soil abiotic and biotic parameters in the upper soil layer (5 to 10 cm) in the control and invaded area (Related to Figure 6, Figure 8 and Discussion). Correlation coefficients and p-values (in brackets) above the diagonal correspond to the ones in the control area, while those below the diagonal are those from the invaded area. Abbreviations are: qO_2 = microbial specific respiratory quotient; BR = Basal respiration; SLA=specific leaf area; LDMC=leaf dry matter content. Correlations between CWMs of plant traits are not shown, as correlation were not performed with the full dataset (see Table S9). Number of observations per area: 9 to 10. Significant correlations ($P < 0.05$) are highlighted in bold, trends ($P < 0.1$) are given in italic.

Soil depth 5 - 10 cm		Control area											
		Soil N	Soil C	Soil pH	BR	Cmic	qO_2	H_2O	CWM Height	CWM SLA	CWM LDMC	CWM Carbon	CWM Nitrogen
Invaded area	Soil N		**0.952 (0)**	0.188 (0.603)	**0.83 (0.003)**	**0.903 (0)**	-0.261 (0.467)	**0.952 (0)**	-0.042 (0.907)	-0.224 (0.533)	**0.697 (0.025)**	0.212 (0.556)	**-0.624 (0.054)**
	Soil C	**0.952 (0)**		0.115 (0.751)	**0.745 (0.013)**	**0.939 (0)**	-0.358 (0.31)	**0.903 (0)**	-0.2 (0.58)	-0.261 (0.467)	*0.576 (0.082)*	0.055 (0.881)	-0.515 (0.128)
	Soil pH	-0.127 (0.726)	-0.236 (0.511)		0.382 (0.276)	0.297 (0.405)	-0.394 (0.26)	0.273 (0.446)	**0.648 (0.043)**	**-0.661 (0.038)**	*0.6 (0.067)*	-0.103 (0.777)	-0.333 (0.347)
	BR	**0.967 (0)**	**1 (0)**	-0.1 (0.798)		**0.758 (0.011)**	0.091 (0.803)	**0.939 (0)**	0.127 (0.726)	-0.333 (0.347)	**0.77 (0.009)**	0.224 (0.533)	*-0.576 (0.082)*
	Cmic	**0.867 (0.001)**	**0.939 (0)**	-0.176 (0.627)	**0.933 (0)**		-0.479 (0.162)	**0.867 (0.001)**	-0.042 (0.907)	-0.406 (0.244)	**0.624 (0.054)**	0.042 (0.907)	*-0.564 (0.09)*
	qO_2	-0.5 (0.17)	*-0.583 (0.099)*	0.35 (0.356)	*-0.583 (0.099)*	**-0.783 (0.013)**		-0.103 (0.777)	-0.067 (0.855)	0.297 (0.405)	-0.176 (0.627)	0.345 (0.328)	0.188 (0.603)
	H_2O	**0.794 (0.006)**	**0.83 (0.003)**	-0.297 (0.405)	**0.883 (0.002)**	**0.758 (0.011)**	-0.367 (0.332)		-0.067 (0.855)	-0.273 (0.446)	**0.733 (0.016)**	0.152 (0.676)	**-0.661 (0.038)**
	CWM Height	-0.285 (0.425)	-0.188 (0.603)	0.03 (0.934)	-0.117 (0.765)	-0.176 (0.627)	-0.167 (0.668)	*-0.576 (0.082)*		/	/	/	/
	CWM SLA	0.273 (0.446)	0.273 (0.446)	-0.224 (0.533)	0.217 (0.576)	0.285 (0.425)	-0.3 (0.433)	0.479 (0.162)	/		/	/	/
	CWM LDMC	**-0.818 (0.004)**	**-0.661 (0.038)**	-0.248 (0.489)	**-0.733 (0.025)**	*-0.552 (0.098)*	0.217 (0.576)	*-0.564 (0.09)*	/	/		/	/
	CWM Carbon	0.358 (0.31)	0.394 (0.26)	0.03 (0.934)	0.417 (0.265)	0.37 (0.293)	-0.267 (0.488)	0.067 (0.855)	/	/	/		/
	CWM Nitrogen	0.37 (0.293)	0.321 (0.365)	0.03 (0.934)	0.267 (0.488)	0.285 (0.425)	-0.183 (0.637)	0.321 (0.365)	/	/	/	/	
